# SiO-induced thermal instability and interplay between graphite and SiO in graphite/SiO composite anode

**DOI:** 10.1038/s41467-022-35769-2

**Published:** 2023-01-11

**Authors:** Ban Seok Lee, Sang-Hwan Oh, Yoon Jeong Choi, Min-Jeong Yi, So Hee Kim, Shin-Yeong Kim, Yung-Eun Sung, Sun Young Shin, Yongju Lee, Seung-Ho Yu

**Affiliations:** 1 Department of Chemical and Biological Engineering, Korea University, Seoul, 02841 Republic of Korea; 2 Advanced Analysis Center, Korea Institute of Science and Technology (KIST), Seoul, 02792 Republic of Korea; 3 School of Chemical and Biological Engineering, Seoul National University, Seoul, 08826 Republic of Korea; 4 Center for Nanoparticle Research, Institute for Basic Science (IBS), Seoul, 08826 Republic of Korea; 5 LG Energy Solution, Research Park, Daejeon, 34122 Republic of Korea

**Keywords:** Batteries, Batteries, Batteries

## Abstract

Silicon monoxide (SiO), which exhibits better cyclability compared to silicon while delivering higher capacity than that of graphite, is an adequate material for the development of lithium-ion batteries (LIBs) having higher energy densities. However, incorporating silicon-based materials including SiO into stable graphite anode inevitably degrades not only cycle life but also calendar life of LIBs, while little is known about their aging mechanisms. Here, SiO-induced thermal instability of the graphite/SiO composite anode is investigated. We reveal that under thermal exposure, SiO accelerates the loss of lithium inventory and concomitantly facilitates the lithium de-intercalation from graphite. This self-discharge phenomenon, which is weakly observed in the graphite anode without SiO, is the result of preferential parasitic reaction on the SiO interface and spontaneous electron and lithium-ion migration to equilibrate the electron energy imbalance between graphite and SiO. Understanding this underlying electron-level interplay between graphite and SiO in the composite anode will contribute toward improving shelf life of SiO-containing LIBs in actual operating conditions.

## Abstract (web summary)

SiO is an adequate anode material for next generation lithium-ion batteries, but little is known about its aging mechanism. Here, the authors report thermal instability of the graphite/SiO composite anode induced by the accelerated loss of lithium from SiO and have elucidated the interplay between graphite and SiO.

## Introduction

For electric vehicles (EVs) to take the dominant position in global automobile market, lithium-ion batteries (LIBs) are required to have higher energy densities^1–3^. Conventional LIBs have used graphite (LiC_6_, 372 mAh g^−1^) as an active material of anode, but its theoretical limit aroused the academic and industrial interest for alternative candidates, such as silicon (Si)^4–8^. Si exhibits almost ten times higher theoretical capacity (Li_3.75_Si, 3579 mAh g^−1^) than that of graphite; however, its drastic volume expansion (~300%) during lithiation lead to particle pulverization, electrical isolation, and unstable ever-thickening solid-electrolyte-interphase (SEI), which results in unacceptable cycle performance^9,10^. Although numerous studies have made efforts to alleviate this issue, their application to the industry remains uncertain^11–13^.

Instead of pure Si, silicon monoxide (SiO) is regarded as a feasible candidate^14^. In return for the lower content of electrochemically active Si, almost inactive Si-O matrix acts as a buffer layer between Si nanodomains, lowering down the volume expansion to ~160%^15,16^. Therefore, the SiO anode shows better cycling stability than the Si anode while delivering a greater theoretical capacity (Li_4.2–4.4_SiO, ~2600 mAh g^–1^ for initial lithiation)^16,17^ than that of the graphite anode. In consideration of the practicality, however, its cycling stability is still insufficient to be used alone as the anode material and thus it can only be utilized as a partial substitute for reliable graphite^18^. Adding SiO into the graphite anode is currently a favorable strategy to increase the cell energy density, and the battery industry has desired to adapt graphite/SiO composite anode to the market. In this situation, the next-generation SiO-containing LIB system should be sufficiently investigated in terms of the real operational conditions^19^.

Commercial LIBs are often exposed to the elevated temperatures, which can severely shorten their shelf life^20,21^. Calendar aging, which describes the time and temperature-dependent performance degradation, is generally attributed to the reactive interface between the anode and the electrolyte and its mechanism has been extensively studied for the conventional LIBs containing graphite anodes^22^. However, for the Si-based LIBs, much attention has been focused on the cycle life whereas their lifespan without cycling remains uncharted. Recent reports exist on the aging of Si-based LIBs under elevated temperatures, but their experimental conditions include cell cycling which can interrupt deconvoluting calendar aging from the entire cell aging^23,24^. In this regard, a comprehensive investigation on the calendar aging mechanism of graphite/SiO composite anode is urgently needed for universal application of the SiO in the battery market. It is expected that in the absence of degradation issues stemming from the volume change during cycling, calendar aging of SiO-containing anodes would also be ascribed to their interfacial reactivity with the electrolyte^25^. Several studies observed the unstable interface of Si-based anodes, but they can only provide the slightest hints to diagnose the calendar aging of SiO-containing anodes^26–28^.

In this study, we focus on the thermal aging of the graphite/SiO anode in comparison with the graphite anode under a charged full cell configuration. Firstly, inferior capacity retention of the graphite/SiO cell compared to the graphite cell is confirmed through the electrochemical experiments. With this result, we observed the accelerated lithium de-intercalation from the graphite in the presence of SiO during high temperature storage via o*perando* optical microscopy and in situ X-ray diffraction (XRD) analysis. To reveal the origin of this phenomenon, we investigated the compositional change occurred on the anode using both scanning and transmission electron microscopy (SEM and TEM, respectively). From the energy dispersive X-ray spectroscopy (EDS) obtained from electron images, we found the intriguing fluorine increase concentrated exclusively on the SiO. X-ray photoelectron spectroscopy (XPS) and scanning transmission electron microscopy-electron energy loss spectroscopy (STEM-EELS) were conducted to reveal the changes in the chemical species of the SEI in detail. At last, we have illustrated a model comprehensively penetrating our observations in terms of the electron-level interplay.

## Results

### Impact of thermal storage on electrochemical performance

**Fig. 1:**
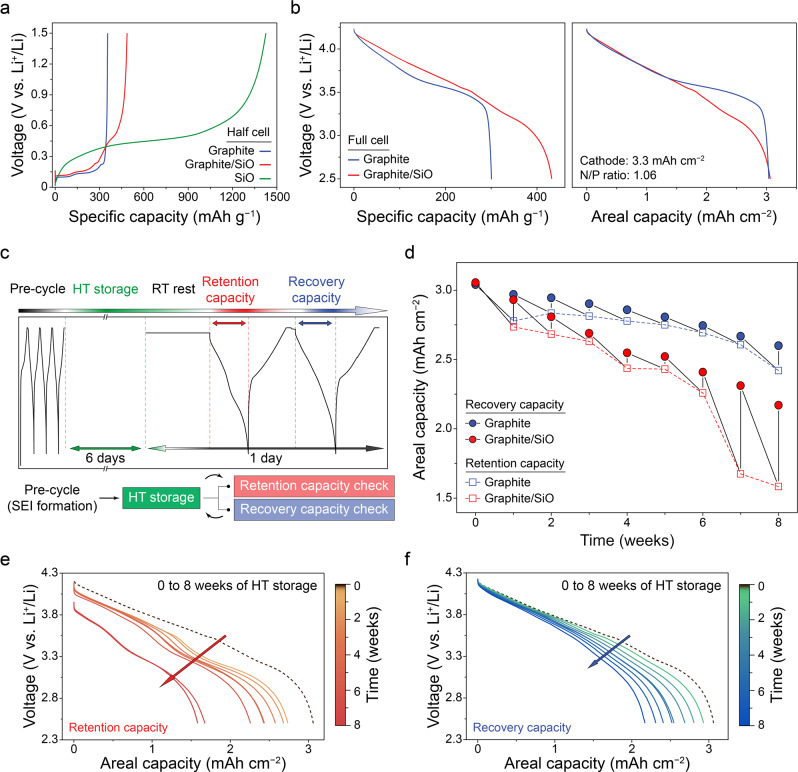
Electrochemical performance and the impact of thermal storage. **a** Voltage profiles of graphite, graphite/SiO and SiO half cells during 3rd de-lithiation. **b** Voltage vs. specific capacity (left) and areal capacity (right) profiles of graphite and graphite/SiO full cells during 3rd discharge. **c** Schematic experimental process of weekly checking capacity values of full cell after periodic HT storage. **d** Decay of retention and recovery capacities measured between periodic HT storage for 8 weeks. The initial value (0th week) indicates 3rd discharge capacity. Corresponding discharge curve variations of **e** retention and **f** recovery capacities of graphite/SiO full cell showing capacity decay compared to the initial one (dashed line).

Figure 1a presents the voltage profiles of the graphite and SiO half cell during de-lithiation. The SiO anode delivered a charge capacity of 1426 mAh g^−1^ (in this paper, g represents the weight of the active material), which is four times higher than that of the graphite anode (355 mAh g^−1^). SiO cannot be utilized alone as an active material for practical use due to its low cyclability compared to that of the graphite. In this respect, we fabricated the graphite-based composite anode containing only 15% of SiO (graphite/SiO anode, 486 mAh g^–1^) to balance the trade-off between cyclability and capacity of the anode. Then we constructed a full cell with the graphite/SiO composite anode and NCM622 cathode (graphite/SiO || NCM full cell) for the following experiments. We also prepared the graphite || NCM full cell as a reference. Figure 1b represents the electrochemical measurements of two lithium-ion full cells with graphite and graphite/SiO anode, respectively. Both cells were fabricated to have an identical areal capacity of cathode (3.3 mAh cm^−2^) and N/P ratio (1.06) in order to specifically evaluate the effect of SiO integration to the graphite anode. The graphite/SiO anode delivered higher specific capacity compared to the graphite anode by 144%, from 300.6 to 432.4 mAh g^−1^ (see Supplementary Fig. 1 for the details on factors affecting the cell performance); in other words, adding 15% of SiO reduces the mass loading of the graphite anode to 70% while delivering identical areal capacity of about 3.05 mAh cm^−2^ (see Supplementary Fig. 2 for an additional note about adjusting the anode areal capacity). The distinct difference in the discharge curves between two full cells, as shown in the right side of Fig. 1b, can be ascribed to the different de-lithiation mechanism on the anode. Since SiO has a higher de-lithiation potential than that of graphite (see dQ/dV curves of the anode half cells shown in Supplementary Fig. 3), SiO fraction in the composite anode electrochemically activates later than that of graphite during discharge, and this is expressed as the gradually diverging voltage curve as much as the SiO participates in delivering capacity.

Incorporation of SiO into graphite anodes earns higher energy density of lithium-ion cells; however, McBrayer et al. reported that Si-based anodes have inferior performance not only in terms of cycle life but also calendar life than that of graphite anodes^20^. We quantified this issue by evaluating the electrochemical performance during periodic storage at a high temperature of 60 °C (termed as HT) at which the rate of the degradation mechanism could considerably increase. Figure 1c schematically depicts this experimental process conducted on the graphite/SiO and the graphite full cells. Before exposed to the elevated temperature, both cells were pre-cycled at 25 °C (room temperature, RT) to form an SEI. The pre-cycled cells were fully charged to the state-of-charge (SOC) 100% and stored at HT for 6 days. Then the cells were rested at RT and cycled twice in a day to measure the capacity values. Here, we named the discharge capacity checked after HT storage as “retention capacity”, and the discharge capacity checked after recharging as “recovery capacity”. To be specific, retention capacity denotes remaining capacity after HT storage, while recovery capacity indicates the capacity that the cell can reversibly deliver. Figure 1d presents the calendar aging of two cells evaluated through repeated experimental processes as mentioned above (see Supplementary Fig. 4 for further details). Both the retention and the recovery capacities of the graphite/SiO cell decayed faster than those of the graphite cell throughout the weeks. Notably, a vast decline of the retention capacity is observed starting from the 7th week. The open-circuit voltage (OCV) of the cell measured after HT storage concurrently dropped drastically (Supplementary Fig. 5).

The series of discharge curves for the retention capacity of the graphite/SiO anode in Fig. 1e clearly shows the aging trend; the capacity decay of the repeatedly recharged graphite/SiO cell during HT storage gradually increases as every week passes, and the transition point appears on the 7th week. However, as shown in Fig. 1f, the recovery capacity of the graphite/SiO cell decayed rather linearly even at the transition point, indicating that the irreversible capacity loss is relatively constant for each period. The transition in the retention and the recovery capacities for the graphite anode shows a similar trend overall, except that the transition point was not observed within the given periods (Supplementary Fig. 6). Presumably, this rapid transition that is observed only in the graphite/SiO anode could be attributed to the gradual decrease of the graphite contribution in the retention capacity of composite anode as HT storage is repeated, which might be induced by the presence of SiO (see Supplementary Fig. 7 for further details). However, we believed that revealing the cause for this transition point appearing in the series of retention capacity is out of the scope in this study. The clear point is that the more the retention capacity is decreased, the more the capacity loss is recovered at subsequent cycle.

On the other hand, the ongoing irreversible capacity loss constantly reduced the performance of the graphite/SiO cell more severely than that of graphite cell. The three-electrode experiments conducted on the same lithium-ion cells (Supplementary Fig. 8) show that NCM cathodes do not reach the electrolyte oxidation potential during charge whereas the anodes are always lower than the electrolyte reduction potential within the operational voltage range; when the recharged cells were put into HT, they were always exposed to the risk of electrolyte reductive decomposition which is known to the main degradation mechanism of the cell aging^22^. Therefore, it is undoubtful that the diminution in the lithium inventory, which is limited in the full cell configuration, have led to the permanent calendar aging of these cells. Usually, the aging of lithium-ion cells is analyzed in terms of three degradation modes: loss of lithium inventory (LLI), loss of anode active material (LAM_anode_), and loss of cathode active material (LAM_cathode_) in the battery field^24,29^. In accordance with this approach, we conducted the coin cell reassembly experiments to diagnose the main cause for the capacity loss among these modes (see Supplementary Fig. 9 for further details). The capacity loss for the graphite and graphite/SiO cells calculated from the capacities of their anodes and cathodes collected from the aged cells (Supplementary Fig. 9b) shows that irreversible capacity loss, which indicates LAM_electrode_, was insignificant compared to the reversible capacity loss which indicates LLI for both full cells; the major degradation modes of the calendar aging of the lithium-ion cells should be attributed to LLI rather than LAM. Furthermore, evaluation of the impact of LLI in the calendar aging to the subsequent cycle aging at RT was conducted (Supplementary Fig. 10). The result shows that the excessive capacity loss after 7 weeks of HT storage occurred and in the subsequent cycles, the rate of capacity decay after the capacity recovered to some extent had no significant difference to that of cycling at RT without storage. This implies that even though LLI occurred during HT storage, the graphite/SiO cell maintained its performance for at least 150 cycles with its reversible lithium. From the above electrochemical performance experiments conducted on the full cells with and without SiO, it can be concluded that thermal aging of the cell is significantly impacted by the composition of the anode. SiO, which can improve the cell energy density, inevitably makes the cells more vulnerable to thermal exposure.

### Accelerated lithium de-intercalation from the graphite

**Fig. 2:**
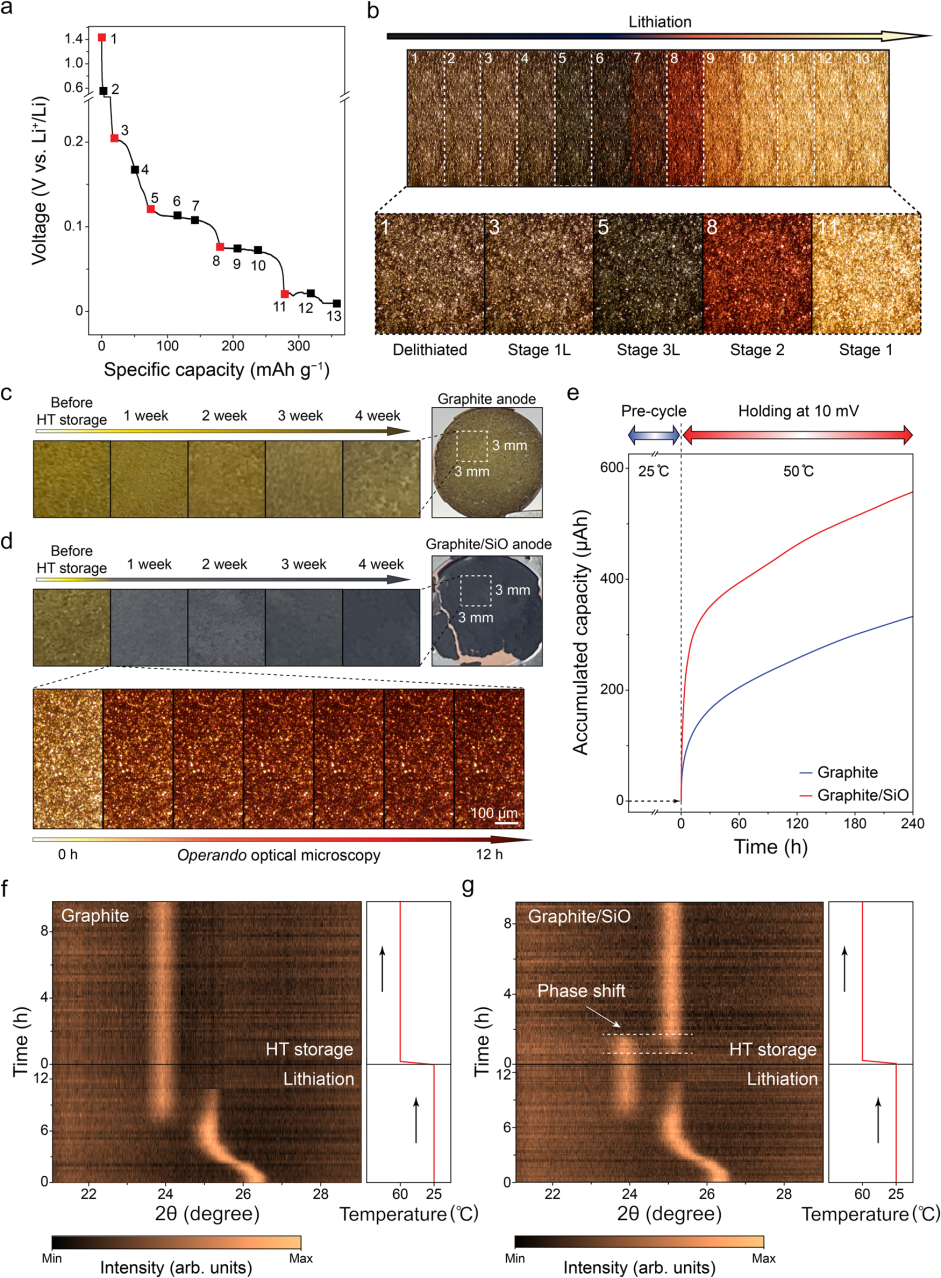
Thermal self-discharge visualized by stage transition of graphite. **a** Voltage profile of graphite half cell during lithiation and **b** corresponding optical images of graphite electrode obtained from *operando* optical microscopy. Among six phases of lithiated graphite (stage 1L, 4L, 3L, 2L, 2, and 1), stage 4L and 2L were not represented since they were indistinguishable. **c**, **d** Postmortem photographs of **c** graphite anode and **d** graphite/SiO anode upon HT storage. *Operando* optical images of graphite/SiO anode upon initial HT storage (lower side of **d**). **e** Accumulated capacities of graphite and graphite/SiO half cells during 10 mV of voltage hold at 50 °C^33^. **f**, **g** In situ XRD of **f** graphite full cell and **g** graphite/SiO full cell during lithiation and subsequent HT storage.

For lithiated graphite, its lithium content can be confirmed intuitively via optical measurements since different stages of Li_*x*_C_6_ (*x* = 0.05–1) have their own characteristic colors^30–32^. Our *operando* optical microscopy experiment conducted for the graphite half cell exhibits the color transition of the graphite electrode upon lithiation. The corresponding voltage profiles and surface images of the graphite electrode are presented in Fig. 2a and b. Starting from the de-lithiated state at point 1, each stage shift of graphite completes at point 3 (stage 1L), 5 (stage 3L), 8 (stage 2), and 11 (stage 1), and the color varies in accordance with the stage shift from black, blue, red, and yellow, respectively. This distinct color change of graphite is a convenient indicator to distinguish the stage transition of graphite in the following experiments.

Figure 2c and d compares the color transitions of the graphite and the graphite/SiO anodes by the postmortem photographs collected from each full cell during HT storage. Both fully charged anodes initially exhibits a yellow color due to the graphite which formed maximally intercalated compounds, LiC_6_. During HT storage, the graphite anode shown in Fig. 2c still remained in stage 1 (yellow) after 4 weeks, indicating that the graphite maintained its lithiated state well. On the other hand, as shown in Fig. 2d, the graphite/SiO anode became darkish after only one week of HT storage, indicating that a rapid transition to stage 2 (red) of the graphite occurred before a week passed. In addition, *operando* optical microscopy for the graphite/SiO full cell was performed to examine the initial transition of the graphite/SiO anode (lower side of Fig. 2d). From the experiment, we observed an immediate graphite phase shift from stage 1 (yellow) to 2 (red) in the graphite/SiO anode within 12 h of HT storage after full lithiation. The results above manifest the accelerated lithium de-intercalation from the graphite in the presence of SiO, and thereby suggests that the interplay between graphite and SiO exists in the composite anode.

When the self-discharge occurs in LIBs, the graphite anode is de-intercalated steadily as the parasitic reaction proceeds through the SEI^33^. As observed in the above optical experiments, the SiO is observed to accelerate the de-intercalation of graphite; the parasitic reaction might have occurred more severely in the anode containing SiO. To verify this, the fully discharged graphite and graphite/SiO half cells were stored at 50 °C and their accumulated capacities were compared by constantly holding their voltages to 10 mV (vs. Li^+^/Li) for 10 days (Fig. 2e). With maintaining the voltage, the measured capacity largely indicates the accumulated parasitic currents passed through the SEI. The rate of parasitic reactions was higher for the graphite/SiO anode compared to graphite anode, which reaffirms that the SiO integration to the graphite contributes to the increase in the self-discharge rate of the anode which is represented as the de-intercalation of graphite.

The stage transition of the graphite during HT storage is also investigated using in situ XRD measurements in Fig. 2f and g. During initial charge, the graphite peaks of both graphite and graphite/SiO full cell gradually shifted from the pristine state (26.3°) to full lithiation state, stage 1 (23.9°). However, after the cells were stored at HT, the graphite peak shifted from stage 1 to stage 2 (25.1°) within 4 h in the graphite/SiO anode, while the peak in the graphite anode remained at stage 1 over 10 h. To reaffirm the difference between the two anodes, ex situ XRD measurements were performed for the fully charged graphite and graphite/SiO anodes before and after HT storage within a range of stage 1 (Supplementary Fig. 11). During 4 weeks of storage, the stage 1 peak in graphite/SiO anode completely disappeared whereas the peak was still observed in the aged graphite anode. The result again shows the evident gap of the stage transition rate between two anodes. As with the previous observation using optical experiments, these diffraction results make it obvious that the incorporated SiO accelerate the lithium de-intercalation from the graphite during calendar aging.

### Interfacial stability of SiO against the electrolyte

From the above experiments, the degree of self-discharge under thermal exposure was evaluated for graphite and graphite/SiO full cells, which was manifested by the capacity loss and the lithium removal from the graphite. Obviously, the incorporation of SiO into graphite-based anode is found to accelerate this phenomenon to a considerable extent. It is widely accepted that the self-discharge process during calendar aging of LIBs mainly derives from the lithium consumption of SEI on the graphite anode^20^ which results in LLI. This aging mechanism seems to occur more in Si-based anodes. Some studies have reported that Si and its SEI are chemically unstable with the electrolyte and thus interfacial reactions persist even in the open-circuit conditions^27,28^. On that account, it is expected that the incorporation of SiO increases the reactivity of anode interface. This increase of the reactivity could facilitate the self-discharge of graphite/SiO full cell system, where the anode is de-lithiated and concomitantly its electrode potential increases. In this regard, OCV variations of graphite and graphite/SiO half cells under 50 °C storage were compared (Supplementary Fig. 12). Both anodes were fully lithiated at RT before being exposed to the elevated temperature. Starting from the voltage of 10 mV, the OCV rises faster in the graphite/SiO than in the graphite half cell. This indicates that the self-discharge and concomitant lithium removal proceeds faster when the anode incorporates SiO, implying the contribution of SiO to the instability of the anode interface.

**Fig. 3:**
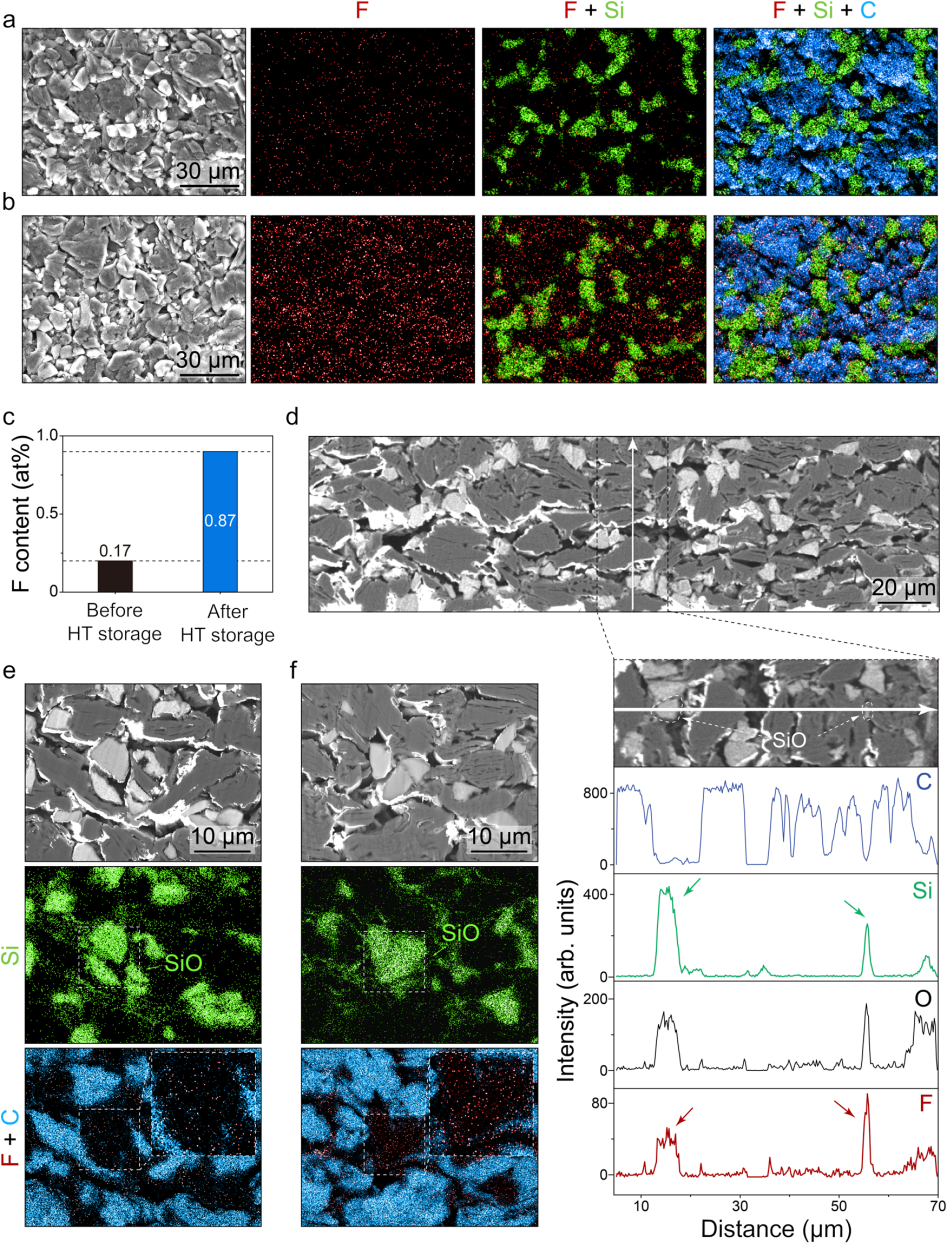
Fluorine increment of the anodes through thermal storage. **a**–**c** Top-view SEM images and EDS elemental maps of graphite/SiO anode **a** before and **b** after 4 weeks of HT storage and **c** corresponding relative atomic ratios of fluorine. **d** Cross-sectional SEM images and line scan profiles of graphite/SiO anode after HT storage. **e**, **f** SEM images and EDS elemental maps of graphite/SiO anode **e** before and **f** after 4 weeks of HT storage.

SEM-EDS analysis was conducted to examine in detail the evolution of anode through exposure to the elevated temperature. Figure 3a and b present the top-view images of the graphite/SiO anodes before and after 4 weeks of HT storage. As shown in elemental maps, fluorine (red) significantly increased throughout the surface as the anodes were exposed to the elevated temperature. Since no fluorine source except LiPF_6_ salt exists in our system and the samples were sufficiently washed, the presence of fluorine indicates the existence of the decomposition products from the LiPF_6_. Thus, it is reasonable to consider that the fluorine content of the anode before storage stemmed from the SEI formation during pre-cycle, while a fivefold increase in the amount of fluorine from 0.17 to 0.87 at% resulted from the decomposition of the electrolyte salt under thermal exposure (Fig. 3c). As it is known that LiPF_6_-containing electrolytes thermally decompose to generate fluorine-containing components on the SEI^34,35^, the fluorine increase can also be observed for the graphite anode. However, the increment was more significant for the SiO anode than the graphite anode (Supplementary Fig. 13). These observations suggest that the SiO in the composite anode facilitates the thermal-induced electrolyte decomposition, which ultimately results in the cell aging accompanying capacity fade and the impedance rise.

Furthermore, we found that the fluorine increases disproportionally among the particles in the composite anode. Figure 3d shows the cross-sectional image of the graphite/SiO anode after 4 weeks of HT storage and the elemental line scan profiles. Along the white line, fluorine (F) greatly increased along the SiO (Si and O) region as pointed by arrows, whereas its intensity is negligible along the graphite (C) region. This noticeable difference clearly indicates that the electrolyte decomposition was concentrated exclusively on SiO particles. Similar results are obtained from the cross-sectional elemental mapping of graphite/SiO anodes before and after 4 weeks of HT storage (Fig. 3e and f). As highlighted by the dashed box, the density of fluorine increases within the SiO region under the elevated temperature. This distinct discrepancy in the interfacial stability between graphite and SiO clarifies that the unstable SiO is responsible for the aggravation of thermal aging. Ha et al. reported that the generation of corrosive hydrofluoric acid (HF) is promoted by the surface oxide layer of Si electrode because the HF in the electrolyte reacts with the surface SiO_2_ to release water, which can hydrolyze LiPF_6_ to regenerate HF^36^. HF can vigorously attack the SEI, provoke the electrolyte reduction, and eventually fluorinate the SEI^20,34^. We presume this phenomenon is also applicable to our study in that we address the SiO having abundant Si–O groups under the harsh conditions.

**Fig. 4:**
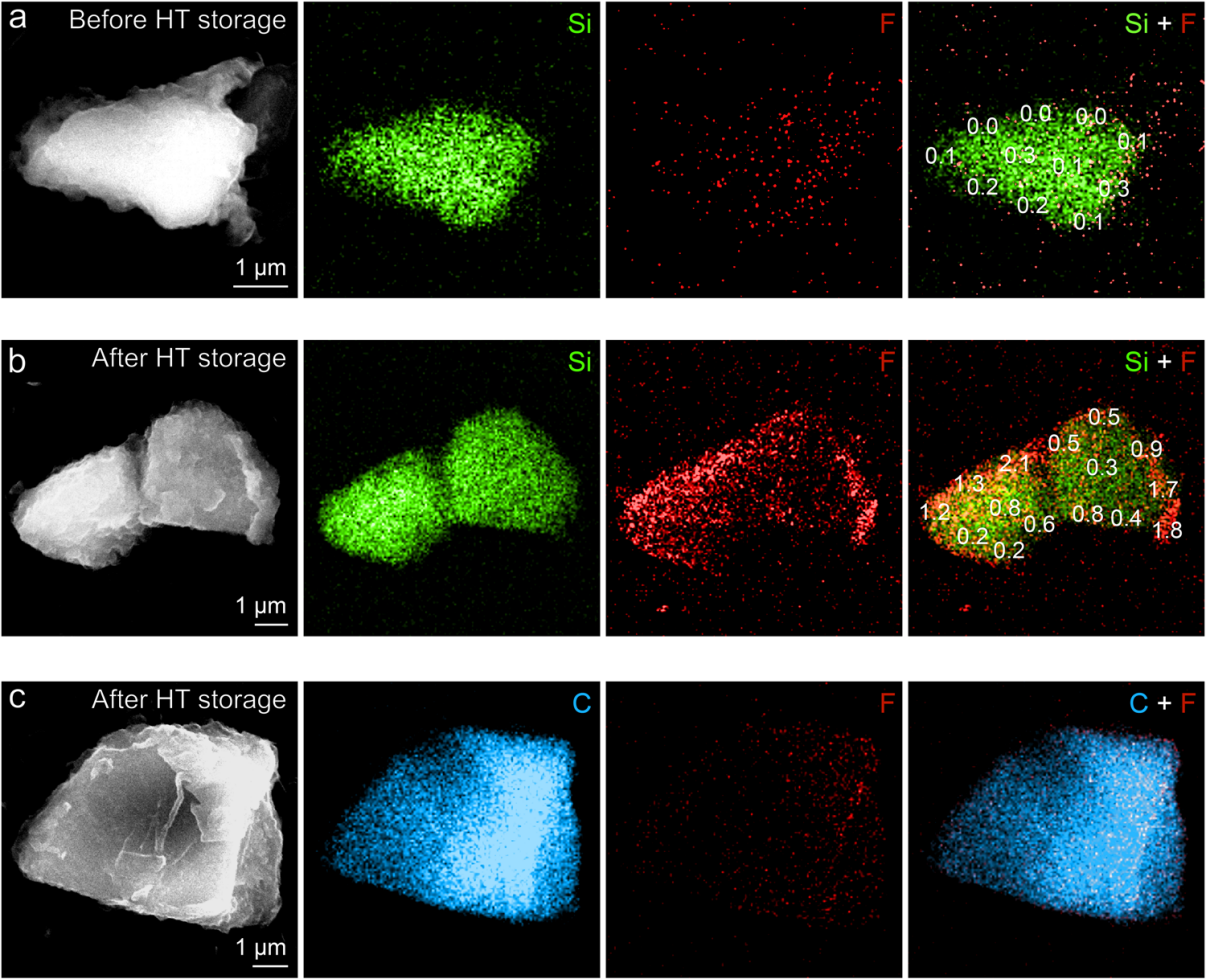
Fluorine increment of the individual anode particles through thermal storage. **a** SEM images and EDS elemental maps of SiO particle collected from graphite/SiO anode before HT storage. **b**, **c** SEM images and EDS elemental maps of **b** SiO and **c** graphite particle from graphite/SiO anode after 4 weeks of HT storage.

Figure 4 presents the SEM-EDS images probing individual particles in the graphite/SiO anode collected before and after 4 weeks of HT storage. The particles were removed from the electrode and scattered on a copper (Cu) grid, allowing the information of a single particle to be reliably obtained. Figure 4a shows the SiO particle obtained from the anode before HT storage, while Fig. 4b and c, respectively, shows the SiO and graphite particle obtained from the same anode after HT storage. As can be observed in the fluorine (red) maps, thermal exposure significantly increased the amount of fluorine on the SiO particle, especially near its surface (Fig. 4a and b). On the other hand, the graphite particle showed negligible intensity of fluorine (Fig. 4c), indicating that the SEI of graphite was rarely affected by thermal exposure. The relative atomic ratios of fluorine for each small fragment of SiO particle are written on silicon and fluorine-overlapped maps (rightmost of the figure) to display the quantitative distribution of fluorine. Before exposed to the elevated temperature, the measured values from all fragments were below 0.3 at%. However, the SiO particle collected after HT storage possesses much higher fluorine content, of which the value ranges from 0.2 to 2.1. These results strongly support our point; since SiO has instable SEI, it inevitably undergoes parasitic processes consuming not only electrolyte but also itself, and accumulates fluorine-containing species on its surface as a consequence, while graphite is well protected from the electrolyte by its rigid SEI in the same situation.

### Compositional change on SEI through thermal storage

When a lithium-ion full cell is at the charged state, the SEI works as an electrical insulating barrier inhibiting thermodynamically favorable electrolyte reduction. However, the metastable components of the SEI can be decomposed during thermal exposure. So far, we found that the SEI of SiO is intrinsically weaker than that of graphite by comparing their intensities of elemental fluorine derived from the electrolyte salt. Although this observation is striking evidence for the relevance between unstable nature of the SEI and thermal aging of the cell, the change in the chemistry of SEI still remains elusive considering its diverse and complex nature. In this regard, further detailed chemical change occurred in SEI was collected using XPS and STEM-EELS, which can probe the top few nanometers of the surface and thus provide SEI-specific information.

**Fig. 5:**
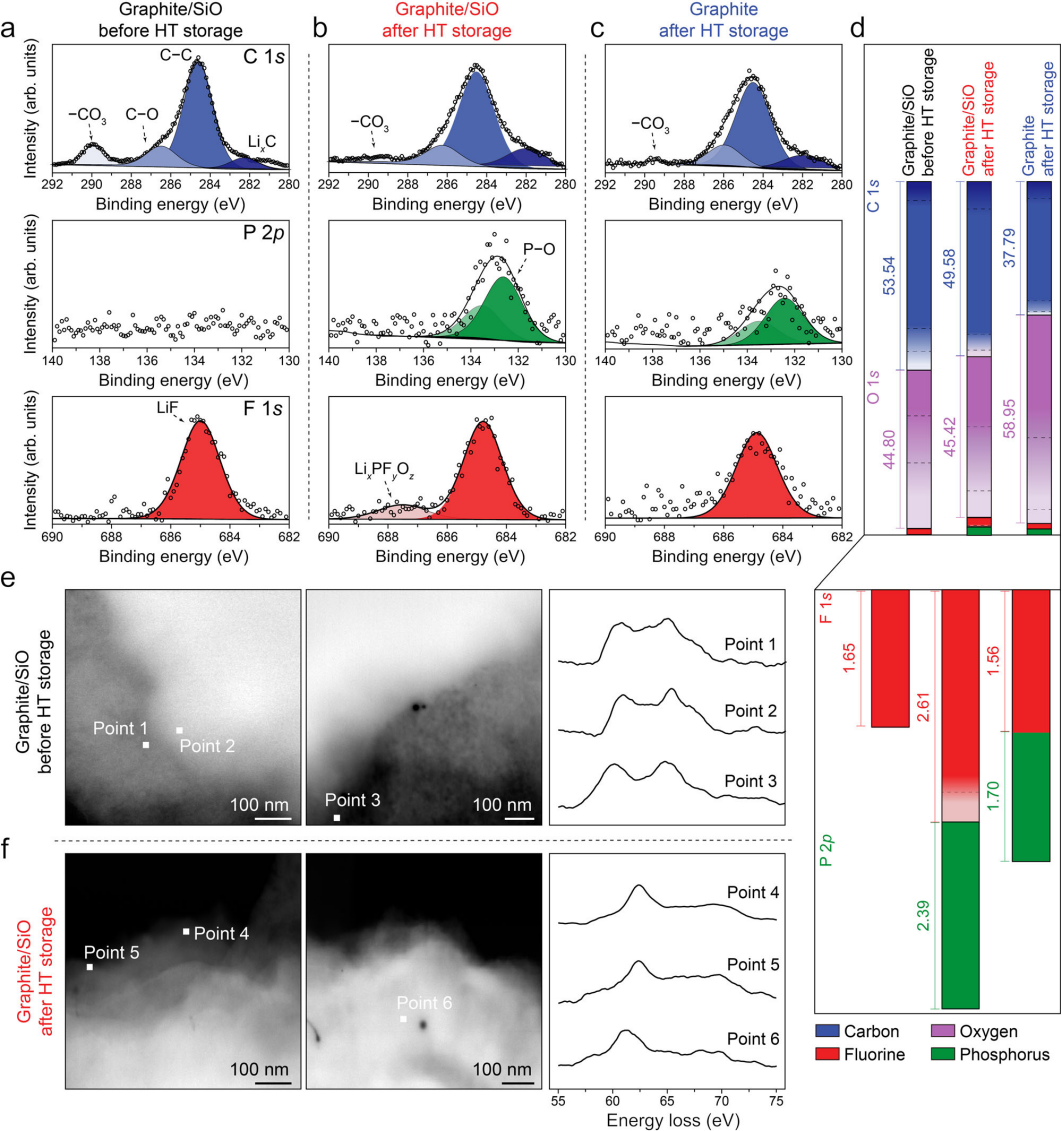
Compositional change on the SEI through thermal storage. **a**–**d** XPS C 1 *s*, P 2*p*, and F 1 *s* spectra of **a** graphite/SiO anode before HT storage, **b** graphite/SiO and **c** graphite anode after 4 weeks of HT storage, and **d** corresponding elemental compositions when the components in four elements (C 1 *s*, O 1 *s*, F 1 *s*, and P 2*p*) were included in the calculation of relative composition. **e**, **f** STEM images and Li K-edge EELS spectra of SiO particles collected from graphite/SiO anode **e** before HT storage and **f** after HT storage.

Figure 5a and b show the XPS C 1 *s*, P 2*p*, and F 1 *s* spectra acquired from graphite/SiO anodes before and after 4 weeks of HT storage (see Supplementary Figs. 14–16 for the other spectra). In C 1 *s* spectra (top), the C–C (284.6 eV) and C–O (286.3 eV) peaks show no significant difference; however, noticeable dissipation of carbonate (−CO_3_, ~289.7 eV) was observed after thermal exposure. Several reports suggested that compared to the inner inorganic-rich layer, the outer organic-rich layer is more likely to be decomposed under thermal aging^28,37,38^. Therefore, it is as the solvent-derived organic species are thermally decomposed, leading to lesser organic nature of the SEI. The considerable decrease of carbonate species (C=O, ~531.8 eV and ROLi, ~530.6 eV) compared to inorganic lithium oxide (Li_2_O, 528.3 eV) as shown in O 1 *s* spectra (Supplementary Fig. 14a) supports this thermal impact on the chemistry of SEI. P 2*p* (middle) and F 1 *s* (bottom) spectra respectively present the occurrence of phosphate (P–O, ~132.5 eV) and decomposed lithium salt (Li_*x*_PF_*y*_O_*z*_, ~687.5 eV) after HT storage, both derived from the LiPF_6_. As confirmed by the previous SEM-EDS analysis, this shows that the decomposition of the electrolyte salt is thermally accelerated and thus the salt-derived inorganic components increase in the SEI^39,40^.

It is worth noting that lithiated graphite (Li_*x*_C, ~282.0 eV) is observed, indicating that the SEI of graphite was thin enough to be detected. On the contrary, no Si 2*p* peak exists (Supplementary Fig. 14b) which implies that the SEI of the SiO is thicker than that of the graphite. This is also observed from the TEM images of both particles collected from the same graphite/SiO anode (Supplementary Fig. 17). However, regarding that no significant difference is observed between the spectra of graphite/SiO and graphite anodes after HT storage as shown in Fig. 5b and c, the discrepancy observed between the thermal aging of graphite and SiO is based only the matter of how much the process progressed.

In Fig. 5d, corresponding elemental compositions are illustrated to compare the quantitative composition of SEI (see Supplementary Fig. 18 and Supplementary Table 1 for further details). The total amounts of C 1 *s* and O 1 *s* components in the graphite/SiO anode only changed slightly during thermal exposure (left and middle); however, along with the results above, the decrease of carbonate species and the increase of lithium oxide is noticeable. In addition, overall F 1 *s* and P 2*p* components which derived from the electrolyte salt relatively increased. Also, the smaller increment in that of graphite anode (right) demonstrates again that in the presence of SiO, the electrolyte salt decomposes more vigorously due to the unstable nature of SiO interface. Here, we excluded the data from Li 1 *s* spectra (Supplementary Fig. 15a) since it is fundamentally difficult to deconvolute the overlapped lithium compounds due to the low intensity and sensitivity of the Li 1 *s* spectra. However, the lithium occupied a considerable amount in all samples when it is included in the calculation of atomic concentration (Supplementary Fig. 15b). This implies that the SEI is abundant with lithium compounds and therefore the lithium chemistry should be considered to reveal the thermal impact on the SEI.

Figure 5e and f present the STEM images and Li K-edge EELS spectra of SiO particles collected from the graphite/SiO anode before and after 4 weeks of HT storage. For each sample, the spectra were carefully obtained from the points located near the topmost surface of SiO particle to specifically detect the SEI. The scans were conducted in several regions to ensure the reliability of the identification. Since the SEI has various lithium compounds, we refer to the experimentally measured spectra in the prior study to differentiate among lithium species^41^. Figure 5e shows the SiO particle before HT storage, and the obtained spectra mostly indicate the presence of Li_2_CO_3_ or Li_2_O at the surface. It should be noted that metastable Li_2_CO_3_ can be easily decomposed to Li_2_O by the irradiated high energy electron beam. As shown in Fig. 5f, the spectra apparently show LiF after HT storage, indicating that the lithium chemistry on the SEI shifts to lesser Li_2_CO_3_ and more LiF. It is reported that carbonate species can react with the decomposition products of electrolyte salt to generate LiF, and this process can result in the transformation of SEI observed above^34^.

## Discussion

**Fig. 6:**
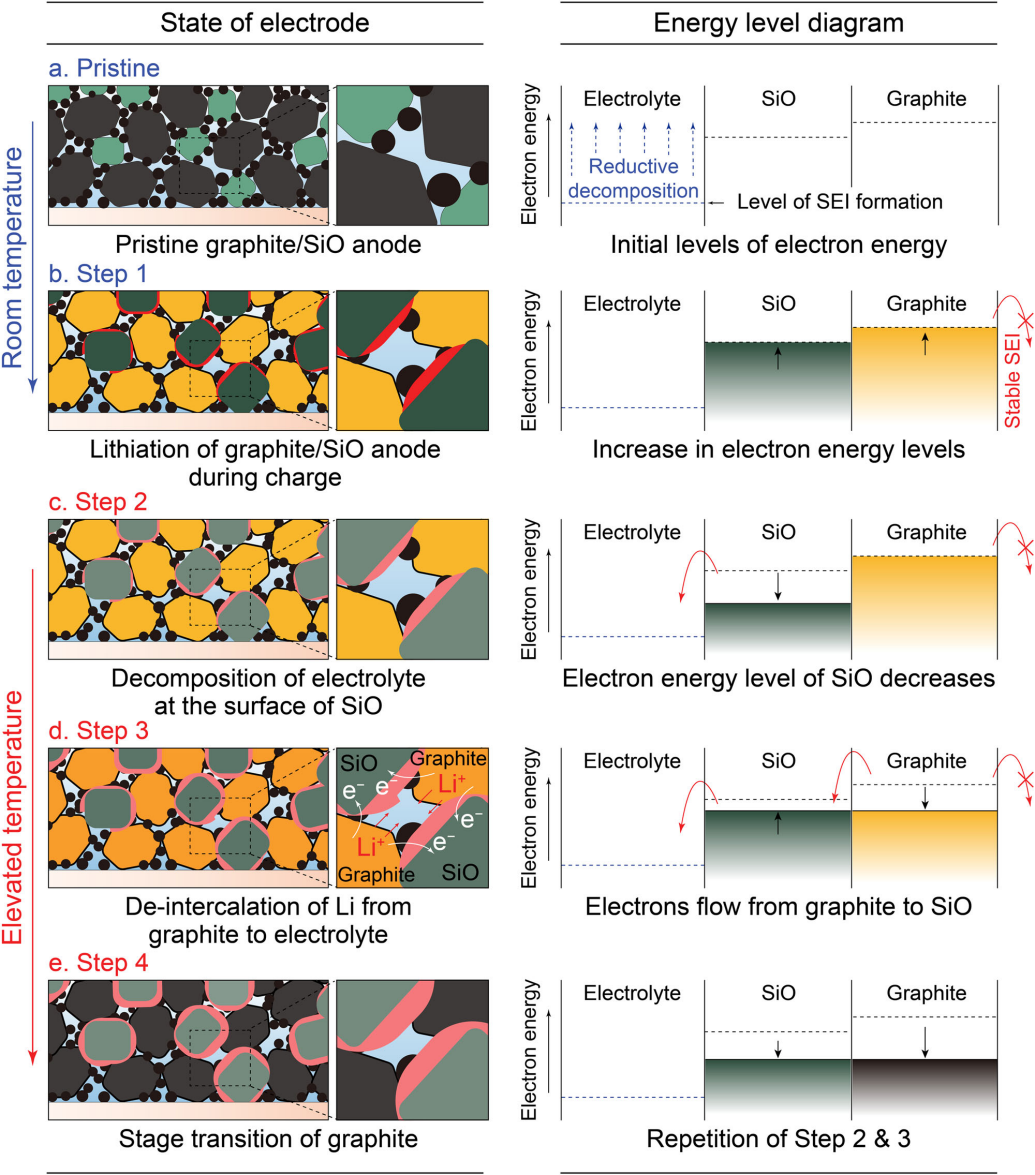
Schematic procedures and relative energy diagrams for each state describing thermal impact on charged graphite/SiO composite anode. **a** Pristine state, fully wetted anode comprised of graphite (gray), SiO (green), and conductive agent (black). **b** Step 1, fully lithiated graphite (yellow) and SiO (light gray) after charging. **c** Step 2, partially de-lithiated SiO with transformed SEI after exposed to the elevated temperature. **d** Step 3, electron movement from graphite to SiO and concomitant lithium-ion de-intercalation from the graphite (orange). **e** Step 4, lithium exhaustion in the graphite (gray) upon prolonged thermal aging.

So far, we applied various techniques to investigate the aging mechanism of SiO-containing lithium-ion cell under the elevated temperature and revealed that the thermal transformation on the SEI is predominant on the SiO, which is the main reason for the lithium consumption from the anode. Based on this, we suggest a comprehensive model for describing the thermal impact on the charged graphite/SiO composite anode as illustrated in Fig. 6. On the left side, the illustrations present each state of the anode upon charging and thermal aging. To elucidate this procedure by means of underlying electron energy, relative electron energy diagrams for each state are presented on the right side. At the pristine state (Fig. 6a), graphite/SiO anode in which the graphite (gray) and SiO (green) are electrically connected directly or via conductive agent (black). This pristine anode is fully wetted with the electrolyte while its level of electron energy is initially under that of SEI formation. During charging at room temperature (Fig. 6b, step 1), initial SEI formation and the lithiation of the active materials gradually occur in the graphite/SiO anode. Within this lithiation process, electrons are electrochemically stored in the anode as the electrode potential decreases; in other words, the electron energy increases. Hence, the electron energy increases simultaneously in both SiO and graphite. After the energy increased to the highest level within the operational range, the graphite/SiO anode became yellow on account of the graphite saturated with the intercalated lithium, while the volume of SiO maximally expanded by forming a lithium alloy.

The aging of this fully charged anode induces self-discharge by interfacial side reaction that consumes stored lithium and results in the decrease of the electron energy level. This self-discharge can occur from both the graphite and the SiO but their rates differ by the stability of their SEI. Unlike the stable SEI on graphite, the SEI formed on SiO is intrinsically unstable and lacks the ability to passivate the electron transfer to the electrolyte. Therefore, when the graphite/SiO anode is stored at the elevated temperature (Fig. 6c, step 2), the side reaction provoked around the SEI of SiO and consequently consumes electrons from the SiO while the graphite is well-passivated by its rigid SEI. Thus, only the electron energy level of SiO decays under the elevated temperature and the instantaneous electron energy gab between SiO and graphite is induced.

This transient electron energy inequality between SiO and graphite is then relaxed by the spontaneous electron transfer (Fig. 6d, step 3). Since active materials are electrically connected, the electron transfer immediately equilibrates this electron energy level imbalance between SiO and graphite. The SiO directly accepts electrons from the graphite while accepting lithium ions from the electrolyte to equilibrate the energy level difference. Simultaneously, since electrons are transferred from graphite to SiO, corresponding amount of lithium is leached from the stable graphite during thermal aging. This energy level balancing of graphite in the graphite/SiO anode is probably comprised of lithium restoring on SiO or assisting the side reaction from the SEI of SiO, since both possibly equilibrate the energy level.

Under the elevated temperature, electrons constantly transfer from graphite to SiO while lithium is leached from the graphite due to the leakage of electrons, which is consumed for the energy level equilibration. After going through the repeated steps described above (Fig. 6e, step 4), the graphite particles eventually become darkish as the lithium drained out even their SEIs are not malfunctioning. The stable SEI formed on graphite is supposed to work as a barrier preventing the leakage of electron energy, and this is the reason graphite anode can maintain its charged state well even in the elevated-temperature condition (Supplementary Fig. 19). However, when the unstable SiO takes part in the construction of anode, the graphite inevitably shares the lithium consumption which stems from the unstable nature of SiO. This unintuitive phenomenon is hard to comprehend with superficial perception of observing phase transition, and can be enlightened with the consideration of the underlying electron-level interplay between graphite and SiO.

In summary, we investigated the thermal impact on the graphite/SiO anode in the charged full cell configuration. The incorporation of SiO increases irreversible capacity loss and self-discharge rate. This degradation originates from the loss of lithium inventory, and SiO accelerates this process by forming a reactive SEI that is vulnerable to the electrolyte. Additionally, in the graphite/SiO composite anode, stable graphite participates in the degradation process to equilibrate the electron energy level difference. This phenomenon is demonstrated by observing the lithium de-intercalation from graphite in graphite/SiO and the preferential side reaction toward SiO, which induces the energy level inequality between graphite and SiO during thermal aging. We suggest a critical perspective on the thermal aging mechanism in the composite graphite/SiO anode, which has been scarcely addressed even though SiO-containing LIBs are on the threshold of industrial application. The deeper understanding of the aggravated thermal aging in the presence of SiO provided in this study will facilitate the establishment of a suitable strategy to gain acceptable long-term stability of SiO-containing LIBs.

## Methods

### Electrode preparation

Industrial-grade graphite, graphite/SiO (graphite:SiO = 85:15 wt%), and LiNi_0.6_Mn_0.2_Co_0.2_O_2_ (NCM622) electrodes were received from LG Energy Solution Ltd. The graphite and graphite/SiO anodes used carbon black and single-walled carbon nanotube (SWCNT) as conductive agents, and sodium carboxymethyl cellulose (CMC) and styrene butadiene rubber (SBR) as binders, and their compositions were limited under 5 wt% to maximize the energy density of the electrode. Likewise, the NCM622 cathode was comprised of NCM622, carbon black, and polyvinylidene fluoride (PVDF) in a weight ratio of 96:2:2. The areal capacity of cathode was 3.3 mAh cm^−2^ and mass loadings of the anodes were adjusted to set N/P ratio to 1.06–1.08. N/P ratios were defined based on the nominal capacity of graphite, SiO, and NCM622 (350, 1350, and 177 mAh g^−1^, respectively).

Laboratory-made electrodes with different compositions were prepared for some experiments as follows and will be specified later. To fabricate the anode, a slurry consisting of active material (graphite and/or SiO, LG Energy Solution Ltd.), super P, CMC, and SBR dissolved in DI water solvent is prepared in an agate mortar. The slurry was casted on a Cu foil using a doctor blade and dried in a vacuum oven at 100 °C overnight. To prepare the cathode, a slurry consisting of NCM622 (MTI Corp.), super P, and PVDF were mixed with N-methyl-pyrrolidinone (NMP) solvent in an agate mortar. The slurry was casted on an aluminium (Al) foil using a doctor blade and dried in a vacuum oven at 120 °C overnight. The electrodes were punched into discs with a diameter of 11 mm. All the electrodes were prepared as above unless otherwise noted.

### Electrochemical measurements

CR2032 coin-type cells (Wellcos Corp.) were assembled in an Ar-filled glovebox. Polyethylene (PE) membrane was used as a separator, and the electrolyte consisting of 1.0 M LiPF_6_ in ethylene carbonate/ethyl methyl carbonate (EC/EMC, 3:7 vol%) with 1.5 wt% vinylene carbonate (VC) and 0.5 wt% 1,3-propane sultone (PS) addtitives (LG energy solution Ltd.) was used as a standard electrolyte (see Supplementary Figs. 20, 21 for the electrochemical performances of other candidate electrolytes). Full cells used NCM622 as a cathode, and half cells used Li metal as a counter electrode. All cells were rested over 12 h at RT before cycled with a WBCS3000 battery test system (WonATech Co., Ltd.).

For the full cell experiments, the cells were charged with constant current (CC) at 0.3 C to 4.25 V, followed by constant voltage (CV) hold until the current decayed to 0.05 C. Then the cells were rested for 20 min and discharged to 2.5 V with 0.3 C CC step. This cycle is repeated three times to form an initial SEI. Pre-cycled cells were fully charged again and stored in open-circuit condition at the elevated temperatures for the target number of days.

Half cell experiments were conducted as follows. To precisely measure the capacity of graphite and SiO electrodes (Fig. 1a) excluding kinetic hindrance, laboratory-made electrodes having low mass loading were used and the cells were cycled at low C-rate (0.05 C). The graphite and SiO electrodes were composed of active material, super P, CMC, and SBR in a weight ratio of 94:1:2:3 and 80:10:4:6, respectively. The mass loading was 2.35 mg cm^−2^ for the graphite electrode, 1.88 mg cm^–2^ for the graphite/SiO electrode, and 0.76 mg cm^−2^ for the SiO electrode. The half cells were discharged with CC at 0.05 C to 10 mV followed by CV hold to 0.01 C, and then rested for 20 min before charged to 1.5 V with a 0.05 C CC step. This cycle is repeated three times to form an initial SEI.

For the 50 °C storage experiments in Fig. 2e and Supplementary Fig. 12, industrial-grade graphite and graphite/SiO electrodes were used. The half cells were discharged with CC at 0.05 C to 10 mV followed by CV hold to 0.01 C, and then rested for 20 min before charged to 1.5 V with 0.05 C CC step. Pre-cycled cells were fully discharged again with the CC-CV step to 10 mV and immediately transferred to 50 °C chamber. After rested in the chamber for 5 min, the cells were moved onto the subsequent steps. For the experiments measuring the accumulated capacities in Fig. 2e, the cells were constantly held at 10 mV. For the OCV measurements in Supplementary Fig. 12, the cells were held at 10 mV to 0.01 C to fully lithiate the electrodes again. Then the cells are rested to measure the OCV variations under the elevated temperature.

### *Operando* optical microscopy experiments

The *operando* optical images were obtained using a customized coin cell with a 3 mm diameter hole sealed with a transparent glass window. The electrodes were directly visualized by optical microscopy (×50 lens, ECLIPSE LV150N, Nikon Corp.), as the same method used in the previous research^42^. For the half cell experiment (Fig. 2a and b), the laboratory-made graphite electrode composed of graphite, super P, CMC, and SBR in a weight ratio of 94:1:2:3 is prepared. The graphite half cell was discharged with CC at 0.1 C to 10 mV followed by CV hold to 0.01 C, and then rested for 20 min before charged to 1.5 V with 0.1 C CC step. For the full cell experiment (lower side of Fig. 2d), industrial-grade electrodes were used. The graphite/SiO full cell was charged with CC at 0.3 C to 4.25 V followed by CV hold to 0.05 C and rested at 60 °C for over 12 h.

### In situ and ex situ XRD experiments

The in situ transmission XRD analysis in Fig. 2f and g was performed using a customized coin cell with a hole sealed with Kapton tape. Cu and Al mesh were employed, respectively, to anode and cathode to minimize interference of the current collector. To utilize the mesh type current collectors, the electrodes were fabricated with relatively high compositions of conductive agents and binders. For the anode, active material, super P, CMC, and SBR were mixed in a weight ratio of 90:5:2:3. For the cathode, NCM622, super P, and polyvinylidene fluoride (PVDF) were mixed in a weight ratio of 80:10:10. The mass loading of cathode was 18.0 mg for the graphite full cell and 19.3 mg for the graphite/SiO full cell, and N/P ratios of both full cells were precisely set to 1.02. The cells were charged with CC at 0.1 C to 4.25 V followed by CV to 0.01 C. After the cycle, the temperature of the chamber immediately increased from 25 °C to 60 °C with a rate of 5 °C/min and the fully charged cells were rested under the elevated temperature for over 9 h. XRD patterns were obtained every 10 min using Smartlab (Rigaku Corp.) with Cu K_α_ radiation (λ = 1.5406 Å) over a 2θ range of 20–30° with a step size of 0.02°. The ex situ reflection XRD analysis in Supplementary Fig. 11 was performed on the anode samples using the same instrument and radiation.

### Material characterization

The fully charged graphite and graphite/SiO full cells were stored at 60 °C for the target number of days. After storage, the cells were disassembled in the Ar-filled glovebox to collect the anodes. Then the anodes were rinsed several times with EC/EMC and then rinsed again with DMC to remove the residual electrolyte. All electrode samples were prepared as above. To prepare particle samples (Fig. 4, Fig. 5e and f, and Supplementary Fig. 17), active material particles were removed from the anode by sonication in EC/EMC and drop-casted onto a lacey-carbon coated Cu grid in the Ar-filled glovebox. Then the grids were rinsed with droplets of volatile DMC to remove viscous EC/EMC and dried overnight.

The top-view scanning electron images and corresponding EDS elemental maps (Figs. 3a, b, and 4) were obtained using field emission scanning electron microscopy (FE-SEM, JSM-7800F Prime, JEOL Ltd.) operating at 15 kV. Six elements (C, O, F, Si, P, and S) were included in the calculation of relative atomic composition. The cross-sectional scanning electron images of the anodes and corresponding EDS elemental line scan profiles and maps (Fig. 3d–f) were obtained using FE-SEM (Regulus 8230, Hitachi Ltd.) operating at 10 kV. In here, the electrodes were cut vertically using ion milling system (IMS, ArBlade 5000, Hitachi Ltd.) under cryo-vacuum condition to minimize ion-beam damage, and immediately transferred to the chamber without air exposure.

XPS (Sigma Probe, Thermo Fisher Scientific Inc.) presented in Fig. 5 and Supplementary Figs. 14–16 was performed with Al Kα (1486.6 eV) at base pressure <10^−9^ mbar. The samples were transferred to the chamber without air exposure. Each spectrum was acquired at pass energy of 30.0 eV and a step size of 0.10 eV. The spectra were processed and fitted using Thermo Avantage software. All spectra were calibrated with C 1 *s* C–C binding energy peak of 284.6 eV. Quantitative compositions were calculated with Scofield’s relative sensitivity factors (RSFs).

TEM operating at 200 kV was performed to obtain the electron images and corresponding EDS elemental maps (Supplementary Fig. 17). The Li K-edge spectra (Fig. 5e and f) were obtained using EELS via Cs-corrected STEM (JEM-ARM200F, JEOL Ltd.) operating at 200 kV.

## Supplementary information

Supplementary Information

## Data Availability

The data that support the findings of this study are available from the corresponding authors upon reasonable request.
