# Supplementary material for: SiO-induced thermal instability and interplay between graphite and SiO in graphite/SiO composite anode
Source: Nat Commun. 2023 Jan 11;14:150. doi: 10.1038/s41467-022-35769-2 (PMC9834238; doi:10.1038/s41467-022-35769-2)
Supplement: Supplementary file 1 — Supplementary Information [file 41467_2022_35769_MOESM1_ESM.pdf]

Supplementary Information

SiO-induced thermal instability and interplay between graphite and SiO in graphite/SiO composite anode

Ban Seok Lee[1,6], Sang-Hwan Oh[1,6], Yoon Jeong Choi[1,6], Min-Jeong Yi[1], So Hee Kim[2], Shin-Yeong Kim[3,4], Yung-Eun Sung[3,4], Sun Young Shin[5], Yongju Lee[5] & Seung-Ho Yu[1]*

[1]Department of Chemical and Biological Engineering, Korea University, Seoul 02841, Republic of Korea

[2]Advanced Analysis Center, Korea Institute of Science and Technology (KIST), Seoul 02792, Republic of Korea

[3]School of Chemical and Biological Engineering, Seoul National University, Seoul 08826, Republic of Korea

[4]Center for Nanoparticle Research, Institute for Basic Science (IBS), Seoul 08826, Republic of Korea

[5]LG Energy Solution, Research Park, Daejeon 34122, Republic of Korea

[6]These authors contributed equally: Ban Seok Lee, Sang-Hwan Oh, Yoon Jeong Choi

*Correspondence and requests for materials should be addressed to S.-H.Y. (e-mail: seunghoyu@korea.ac.kr)

Inventory of Supplementary Information:

Supplementary Notes for Supplementary Figure 7 and 9

Supplementary Figures 1–20

Supplementary Table 1

Supplementary References (1–6)

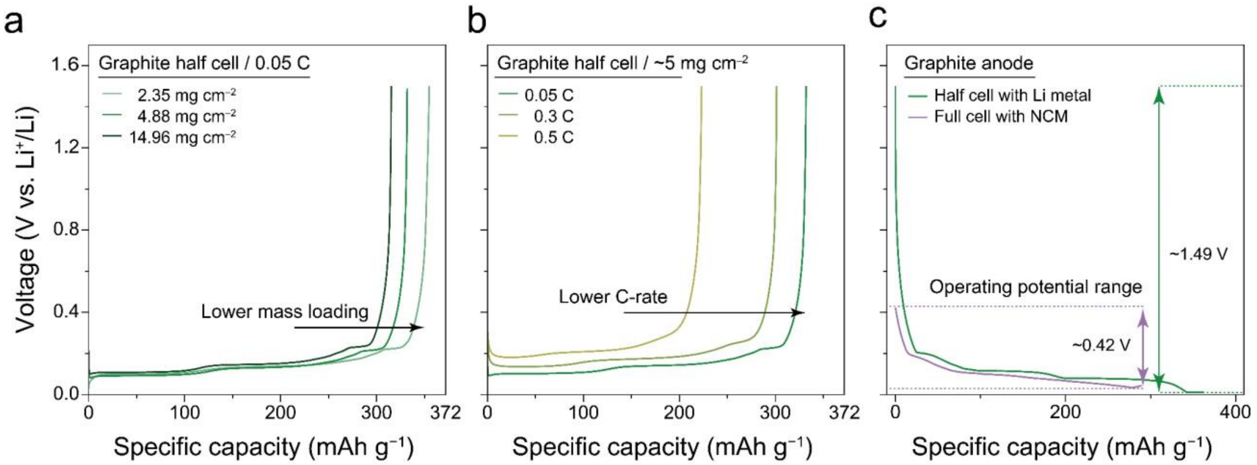

Supplementary Figure 1. Voltage profiles of graphite half cells at 3rd de-lithiation **a** with different mass loadings (each 2.45, 4,88 and 14.96 mg cm^{-2}) and **b** C-rates (each 0.05 C, 0.3 C, 0.5 C), indicating that the lower the mass loading and the C-rate, the closer the capacity of graphite anode can reach to the theoretical maximum (372 mAh g^{-1}). **c** Comparison of operating potential range of graphite anode when it is tested under half cell with Li metal or full cell with NCM cathode (see corresponding data from three-electrode cell shown in Supplementary Fig. 8), showing that the graphite anode operates within much narrower potential range and thus cannot fully activated.

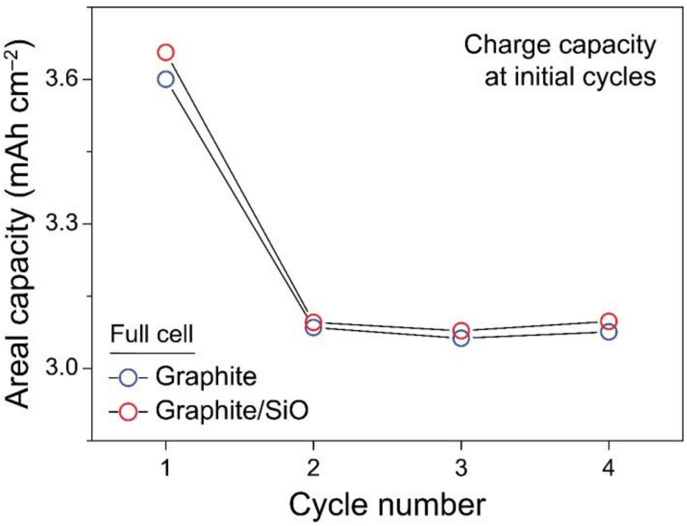

Supplementary Figure 2. Charge capacity of graphite and graphite/SiO full cells at initial cycles. The mass loadings of both anodes are optimized to deliver the identical discharge capacity of 3.05 mAh cm^{-2} within initial cycles. However, since SiO irreversibly consumes lithium to form lithium silicate phases at its initial cycles[1] and induces faster capacity loss compared to the graphite, the charge capacity was generally higher in the graphite/SiO cells than in the graphite cell at their initial cycles.

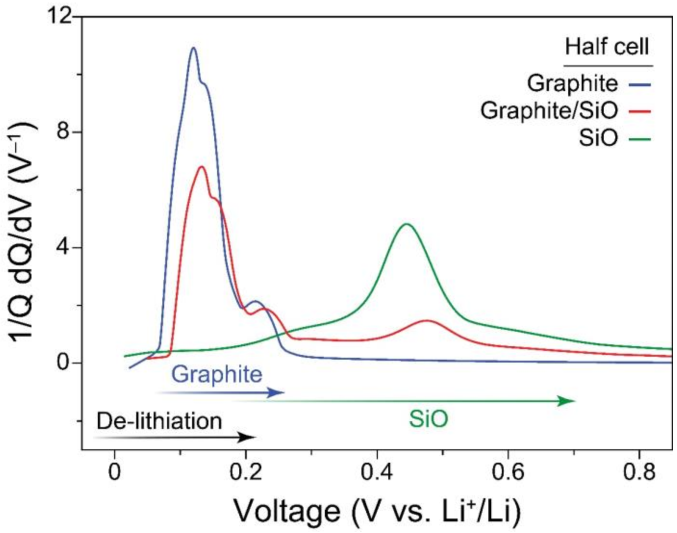

Supplementary Figure 3. dQ/dV curves for de-lithiation of the graphite (blue), graphite/SiO (red), and SiO (green) half cells (see corresponding voltage profiles in Fig. 1a). The plots were normalized by the capacity of each anode. Graphite peaks appeared rather sharply between 0.05–0.25 V while SiO showed a broader peak between 0.2–0.8 V.

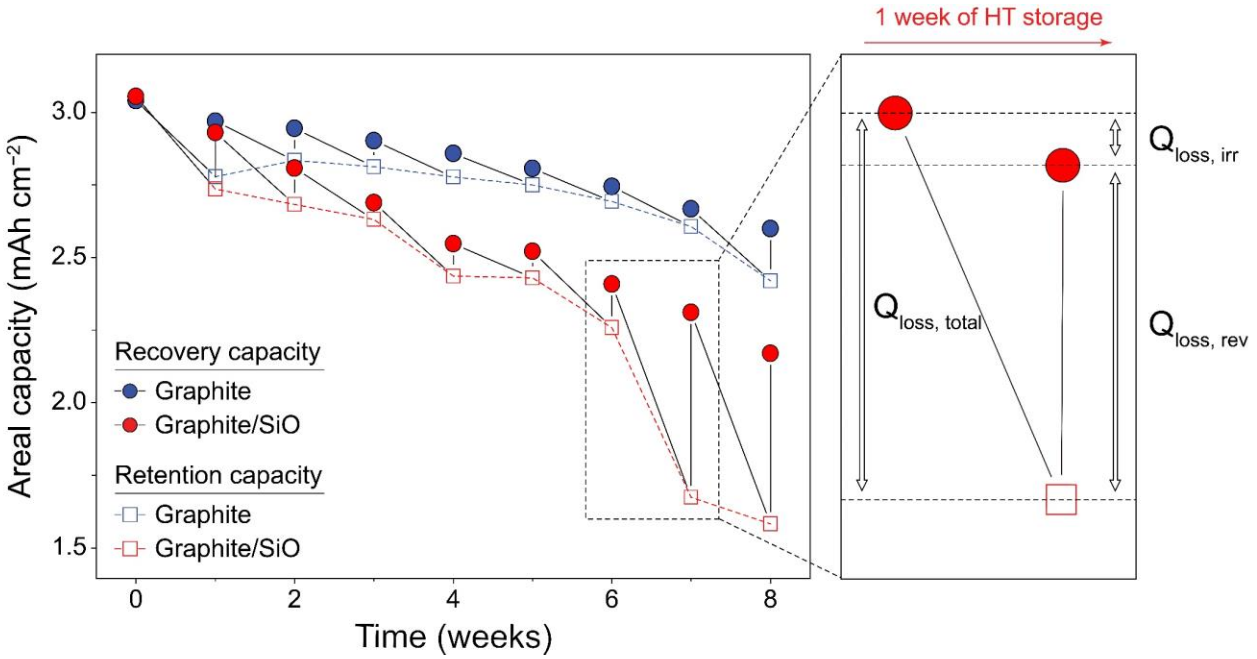

Supplementary Figure 4. Detailed explanation for a single period of HT storage in Fig. 1d. Before HT storage, the cell was pre-cycled followed by a last charge. The 3rd discharge capacity is set as the initial standard value ($Q_{before,\ initial}$) of the cell, and for the other periods, each recovery capacity is set as the standard value (Q_{before}) to evaluate the capacity loss (Q_{loss}) of the following week. During HT storage, the charged cell was self-discharged and some of the lithium might have been irreversibly consumed through the side reactions by which the cell has lost its lithium inventory. After 1 week, the aged cell was discharged to check the capacity remaining in the anode, which we termed as retention capacity ($Q_{retention,\ after}$), and gained the total capacity loss ($Q_{loss,\ total} = Q_{before} - Q_{retention,\ after}$). Then the cell was recharged and followed by the discharge step to check the reversible capacity, terming it as recovery capacity ($Q_{recovery,\ after}$). Now, the irreversible capacity loss ($Q_{loss,\ irr} = Q_{before} - Q_{recovery,\ after}$) is obtained, and much of the capacity loss was recovered in which the amount is denoted as reversible capacity loss ($Q_{loss,\ rev} = Q_{loss,\ total} - Q_{loss,\ irr}$)[2].

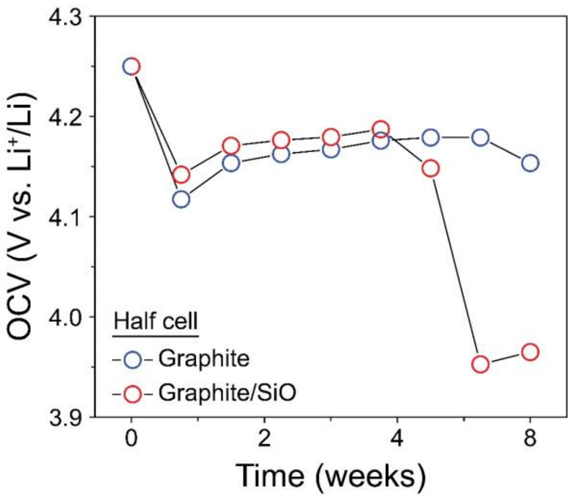

Supplementary Figure 5. OCV variations of graphite and graphite/SiO full cells during periodic HT storage (corresponding to the experiments in Fig. 1d). The values were measured every week at RT rest after HT storage. The drastic decay of about 0.2 V for the graphite/SiO cell appeared at the 7th week, while the graphite remained almost unchanged for 8 weeks.

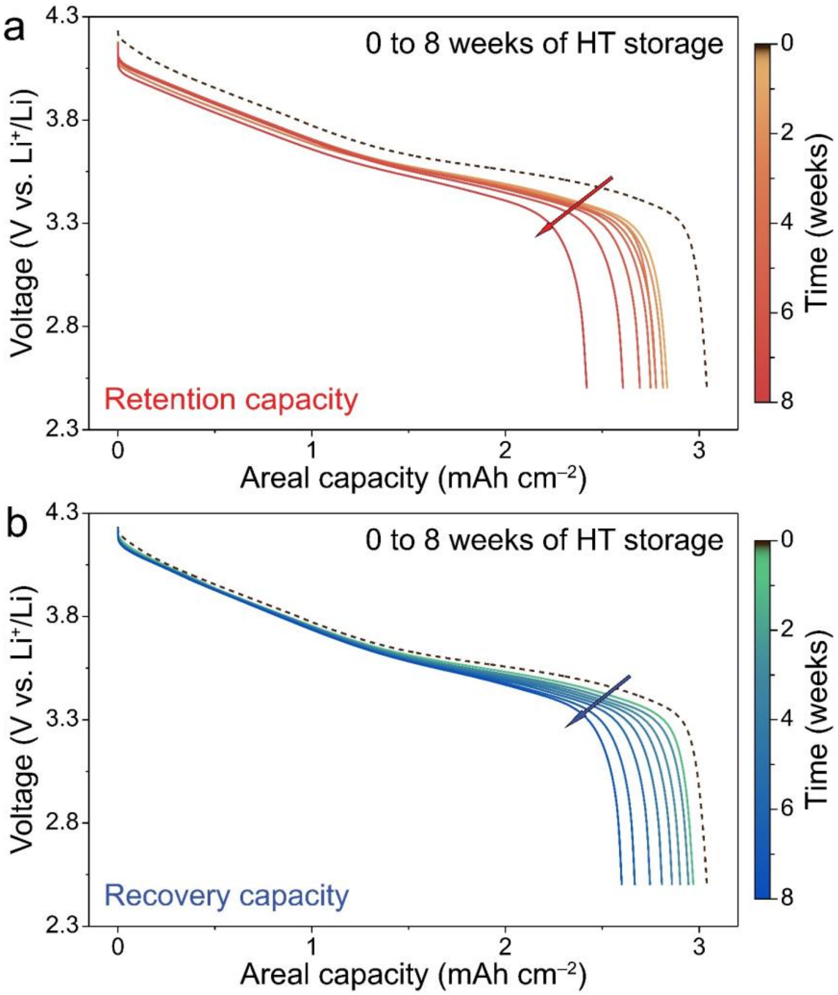

Supplementary Figure 6. Discharge curve variations of **a** retention and **b** recovery capacities of graphite full cell for 8 weeks of periodic HT storage showing capacity loss compared to the initial curve (dashed line). The profiles were obtained through the experimental process shown in Fig. 1c.

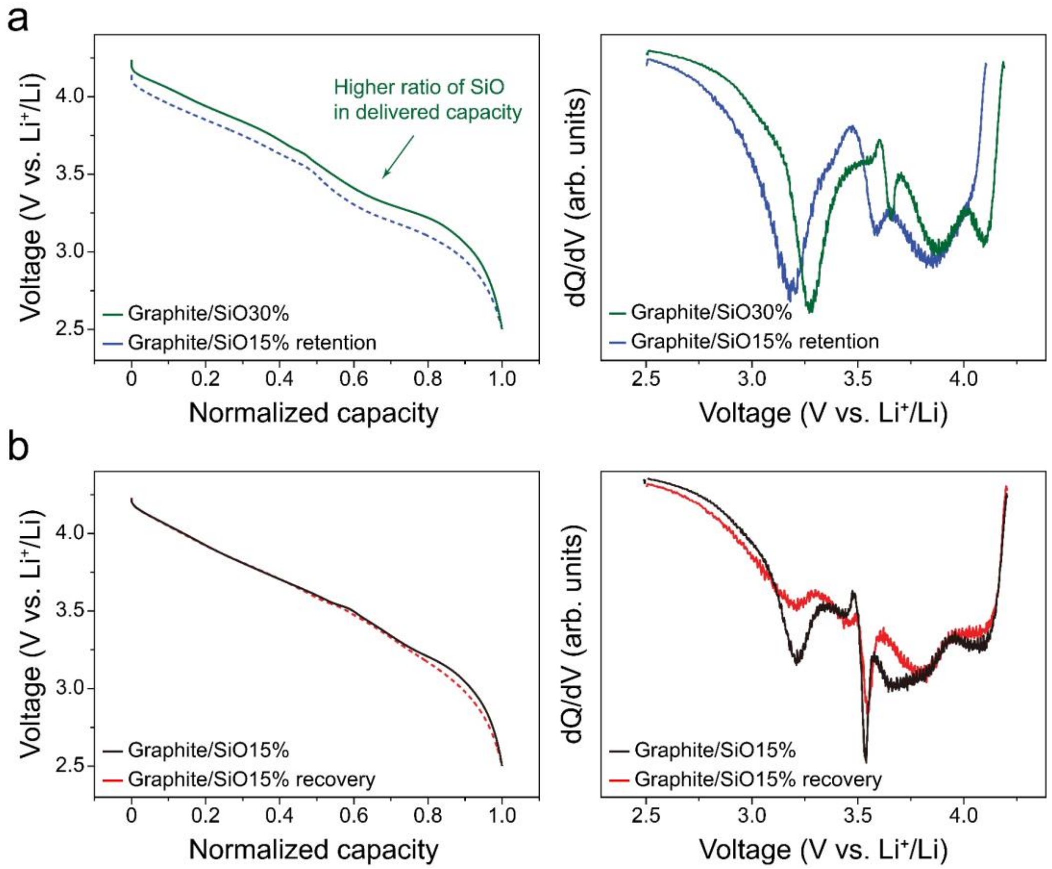

Supplementary Figure 7. Electrochemical analysis investigating difference of discharge curves between retention and recovery capacity check-ups. Voltage profiles and corresponding dQ/dV curves of **a** graphite/SiO15% full cell at retention capacity check after 1 week of HT storage and graphite/SiO30% full cell at initial cycle (3rd discharge), and **b** graphite/SiO15% full cell at recovery capacity check after 1 week of HT storage and graphite/SiO15% full cell at initial cycle (3rd discharge). The profiles were plotted versus normalized capacity for easier comparisons.

Supplementary Note for Supplementary Figure 7.

Methods. A composite anode with higher SiO content of 30% (graphite/SiO30% anode) was fabricated and its composition and areal capacity was same as that of industrial-grade anodes as explained in the Methods section. Graphite/SiO30% || NCM coin full cell was constructed with the same configuration and the electrochemical measurements were performed with the same procedure for the full-cell experiments as explained in the Methods section.

Discussion. The 3rd discharge profile of graphite/SiO30% was compared with the retention capacity profile of graphite/SiO cell (denoted as graphite/SiO15% to avoid confusion) as shown in Supplementary Fig. 7a. Aside from the impact of overpotential which was higher in the retention graphite/SiO15%, both of their profiles and corresponding dQ/dV curves showed similar behavior, which shows that during retention capacity check after 1 week of HT storage, graphite lost its lithium content more than that of SiO during HT storage. This is as graphite has lower electrode operating potential than that of SiO (see Supplementary Fig. 3), de-lithiation during calendar aging would proceed in the graphite prior to the SiO in the composite anode. On the other hand, the recovery capacity profile (and the corresponding dQ/dV curve) of the graphite/SiO cell is similar to that of 3rd discharge profile of graphite/SiO cell before storage, since the cell is recharged and thus both graphite and SiO would have lithiated fully before the recovery capacity check.

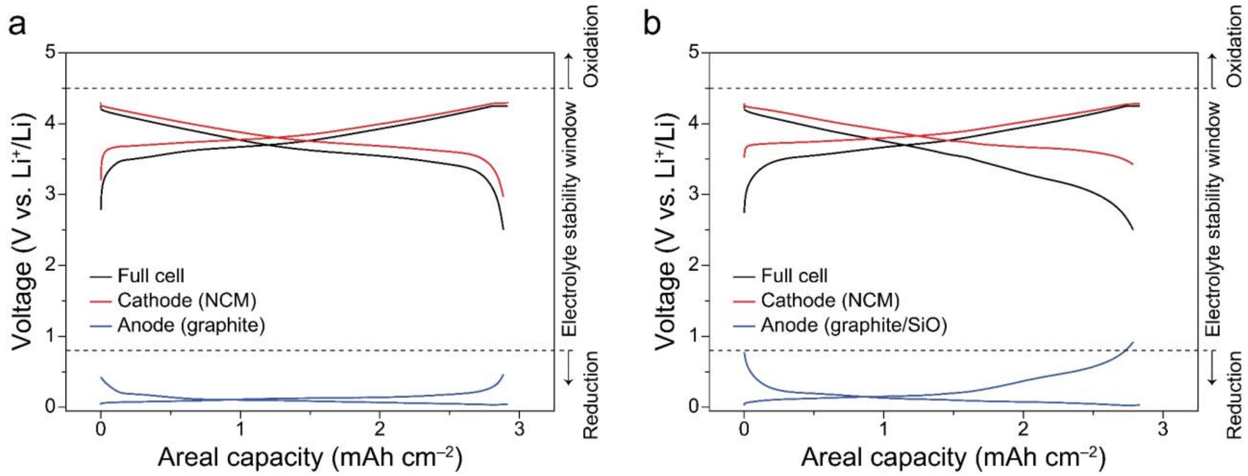

Supplementary Figure 8. Voltage profiles of three-electrode graphite and graphite/SiO full cell. Both cathodes do not reach the electrolyte oxidation potential whereas the anodes are always lower than the electrolyte reduction potential within operational voltage range. The three-electrode cell was fabricated by referring to the assembly method conducted in the previous study[3].

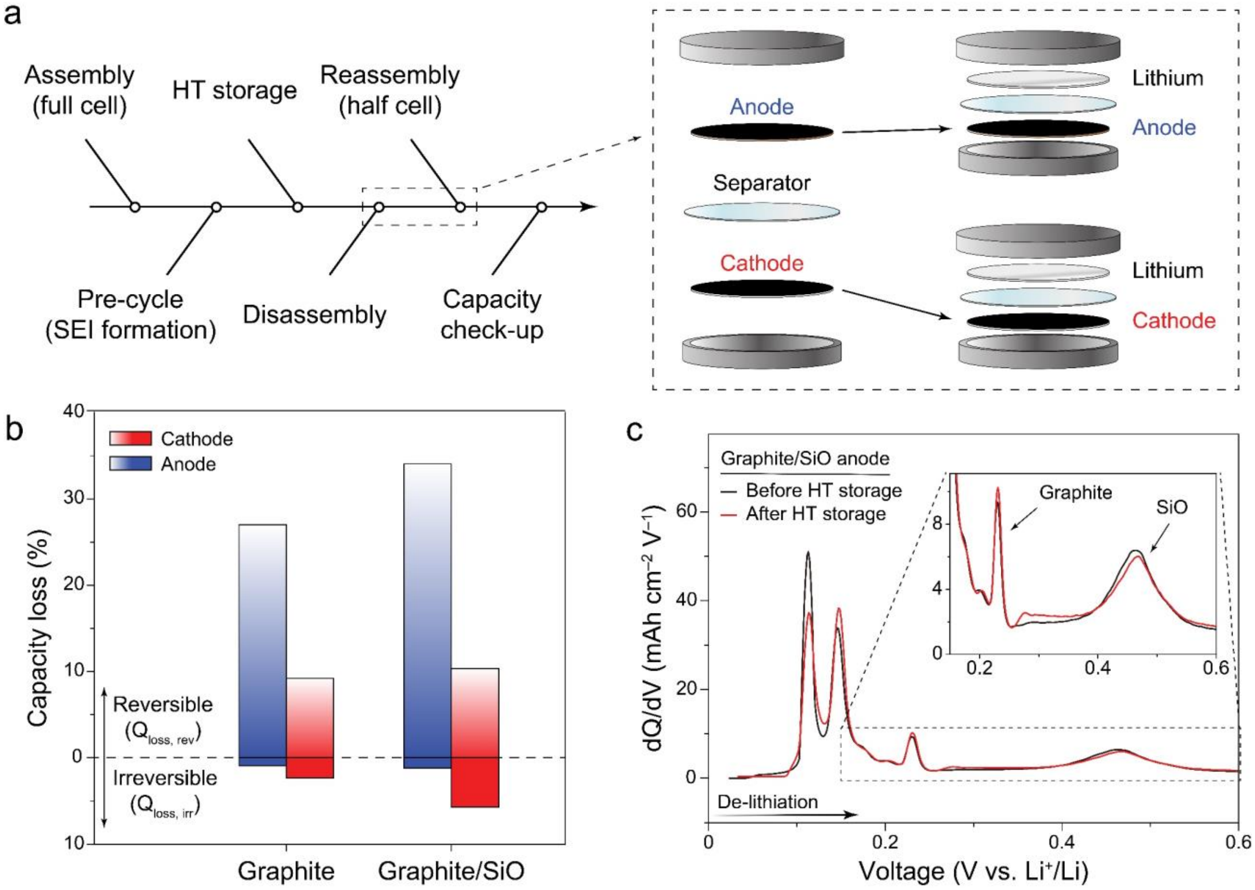

Supplementary Figure 9. a Procedures of the coin cell reassembly experiments. **b** Capacity loss of anodes and cathodes collected from graphite and graphite/SiO cells after 4 weeks of HT storage. The reversible and irreversible values ($Q_{loss, rev}$ and $Q_{loss, irr}$) were calculated from the equations provided in Supplementary Fig. 3. **c** dQ/dV curves for the profiles of graphite/SiO anode half cell at recovery capacity check before and after HT storage.

Supplementary Note for Supplementary Fig. 9.

Methods. Firstly, the assembled graphite and graphite/SiO full cells were pre-cycled (3rd discharge capacity is set as initial value, $Q_{initial,\ full}$). Then the cells were aged at HT, carefully disassembled after 4 weeks, and reassembled into the anode and cathode half cells. Then, similar to the experimental process in Fig. 1c, the half cells were cycled with two times of CC de-lithiation and CC-CV lithiation with CV hold to 0.01 C in order to check the retention and recovery capacities. It is worth mentioning that we chose 0.05 C, far slower than 0.3 C applied for the pre-cycle step, as the measuring current to eliminate the kinetic hindrance which disturbs the precise determination of the degradation modes (LLI, LAM, etc.) of the cells. This method is termed as "quasi-stationary or pseudo-OCV measurement"; a well-used procedure in the battery studies that focus on probing the aging mechanism of lithium-ion cells[4,5]. The voltage ranges were 1.5–0.01 V for the anode half cells and 2.5–4.25 V for the cathode half cells. Same measurements were conducted for the reference cells which were reassembled right after the end of pre-cycle (denoted as before HT storage), and used to normalize the capacity loss to exclude any unavoidable variables such as influence that may have been caused during the cell reassembly on the electrodes, and the different operating voltage ranges and currents.

Calculations. At the initial de-lithiation steps of the half cells (charge for the anode and discharge for the cathode), the retention capacities ($Q_{retention,\ half}$) and the total capacity loss ($Q_{loss,\ total} = Q_{initial,\ full} - Q_{retention,\ half}$) were obtained. Likewise, subsequent de-lithiation step provides the recovery capacities and the irreversible capacity loss ($Q_{loss,\ irr} = Q_{initial,\ full} - Q_{recovery,\ half}$) (see Supplementary Fig. 4 to aid the understanding of this calculation). We postulated that the LAM is quantified by the irreversible capacity loss, and the remaining portion in the total capacity loss indicates the LLI, based on the approach investigating the degradation modes which is well documented in the references[5,6]; LAM is indicated by the "narrowing of the electrode SOC window" which can be identified by the capacity check-up through the pseudo-OCV measurement, and LLI is indicated by the "shifting of the electrode SOC window".

Discussion. The reassembly experiments were conducted to investigate the degradation modes (LLI, LAM, etc.) of our system and elucidate whether the loss of active material (LAM) exists in these electrodes. As shown in Supplementary Figure 9b, the capacity loss during 4 weeks of HT

storage is greater in the anode than of that in the cathode for both graphite and graphite/SiO full cells. Moreover, in accordance with the experiments in Fig. 1d, the total capacity loss is more severe in the graphite/SiO anode (35.3%) than in the graphite anode (27.9%). However, the irreversible capacity loss is rather insignificant compared to the reversible capacity loss in both anodes; it is speculated that LAM is not the major degradation mode in our study. dQ/dV curves for the recovery capacity profiles of graphite/SiO anode half cell before and after HT storage (Supplementary Fig. 9c) provide more direct evidence on the minor contribution of LAM. Both graphite and SiO de-lithiation peaks remained almost unchanged, indicating that the entire anode active material is still active, thereby revealing that LLI predominantly causes the self-discharge during HT storage.

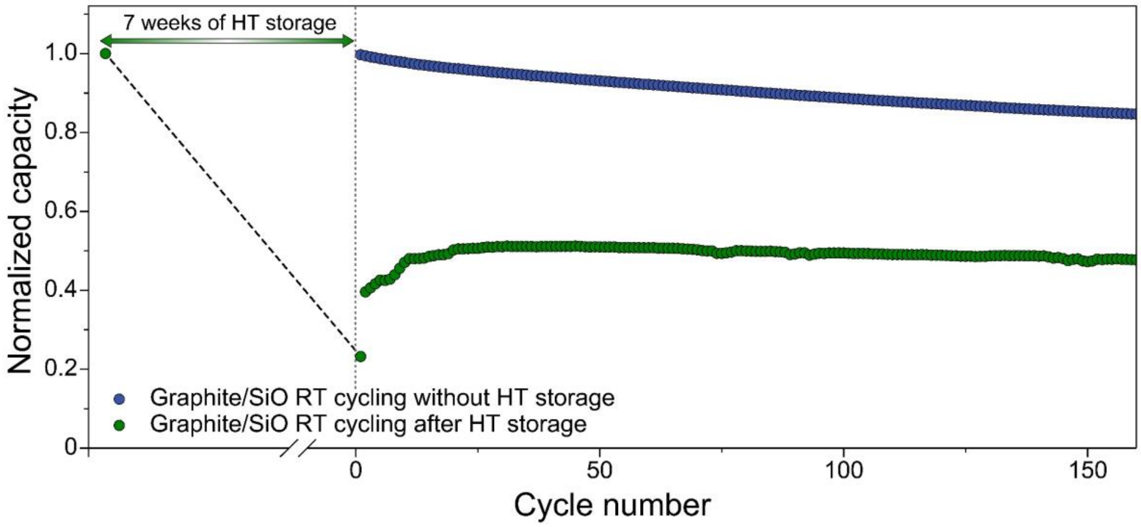

Supplementary Figure 10. Normalized discharge capacity of graphite/SiO full cell cycled at RT without and after 7 weeks of HT storage. Each 3rd discharge capacity is set as the standard value.

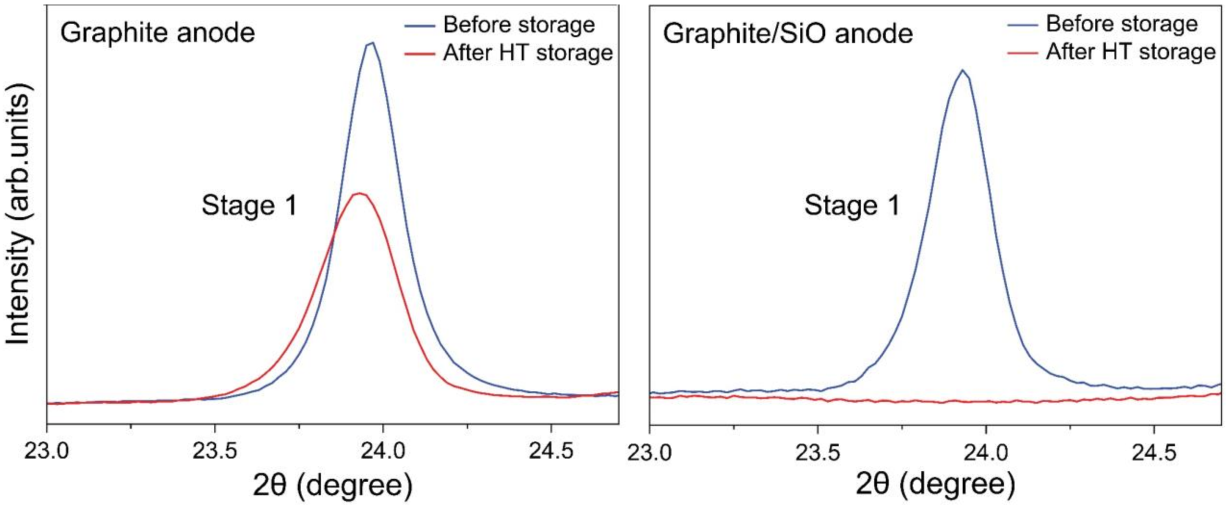

Supplementary Figure 11. *Ex-situ* XRD of graphite anode and graphite/SiO anode before and after 4 weeks storage collected from the full cells.

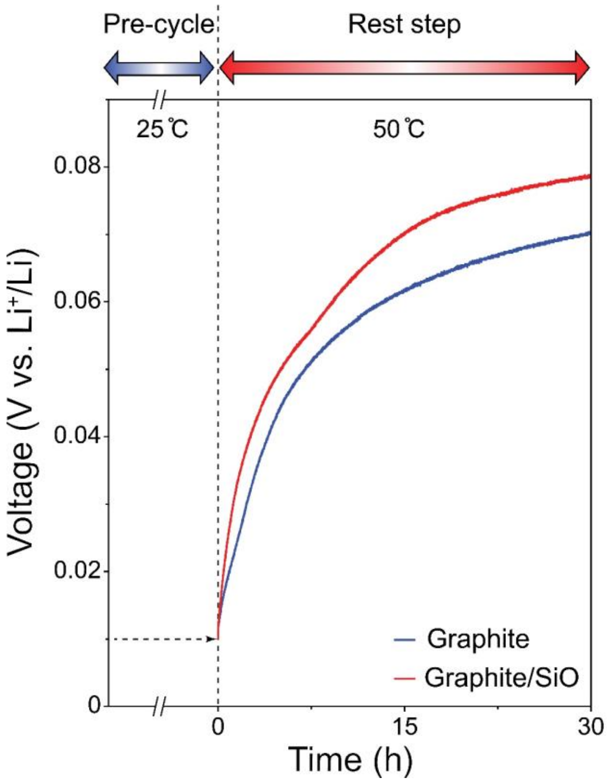

Supplementary Figure 12. OCV variations of graphite and graphite/SiO half cells during RT rest under 50 ℃ storage after pre-cycle.

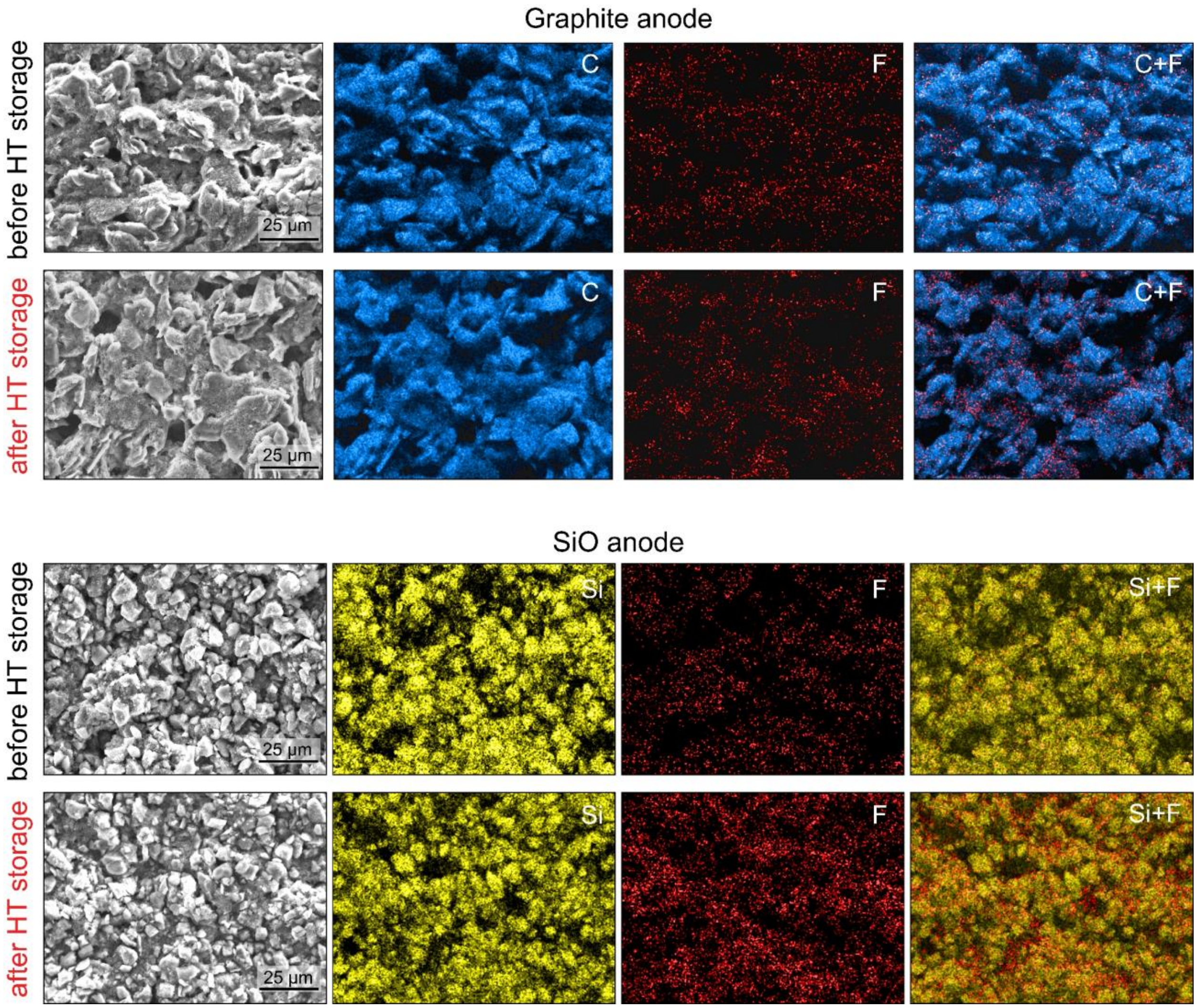

Supplementary Figure 13. Top-view SEM images and EDS elemental maps of graphite anode (top) and SiO anode (bottom) before and after 4 weeks of HT storage. Graphite and SiO anodes used in here were composed of active material, super P, CMC, and SBR in a weight ratio of 90:5:2:3 and 80:10:4:6, respectively.

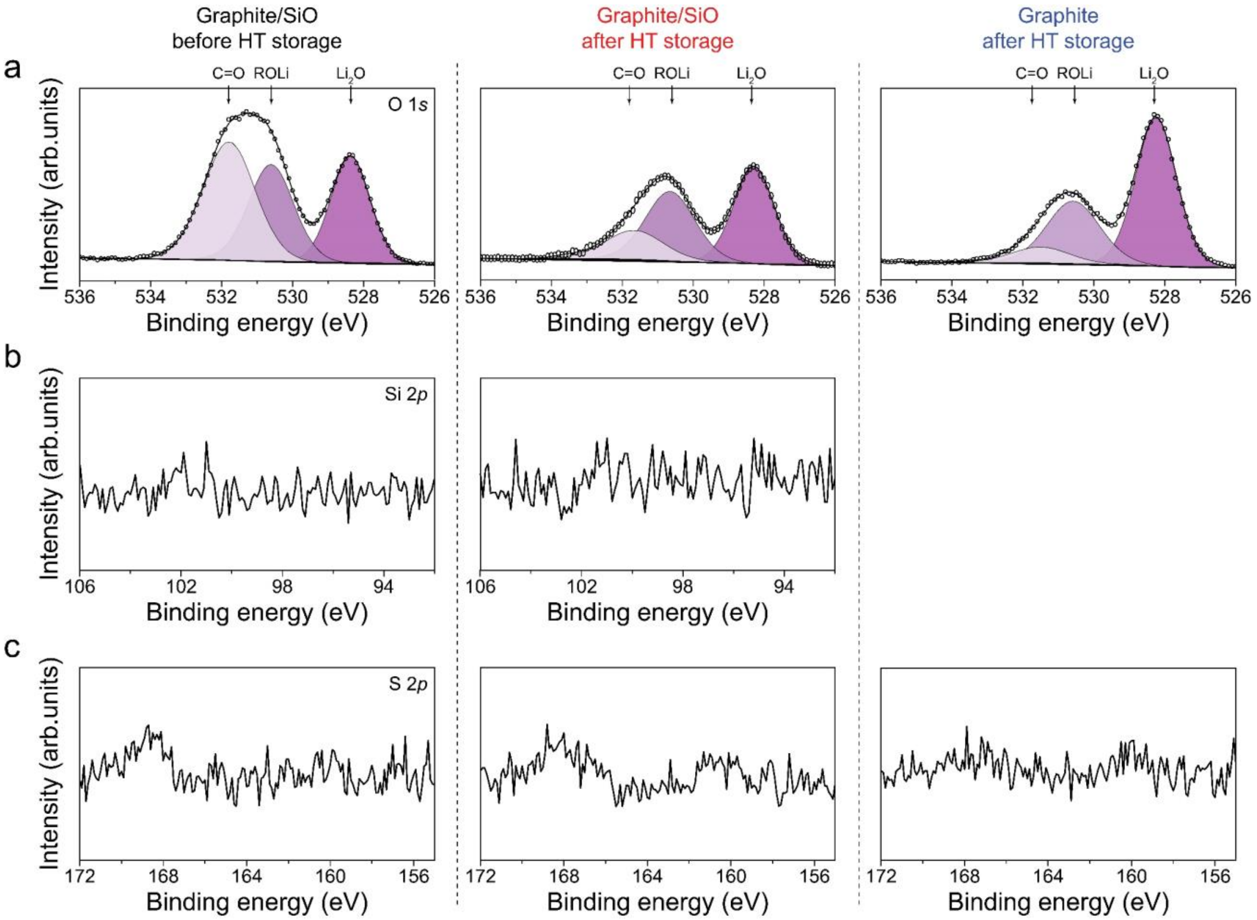

Supplementary Figure 14. XPS **a** O 1*s*, **b** Si 2*p*, and **c** S 2*p* spectra of graphite/SiO anode before HT storage, graphite/SiO and graphite anodes after 4 weeks of HT storage. In O 1*s* spectra, three peaks (C=O, ROLi, Li_2O) are observed for all samples and they were included in calculating the elemental compositions in Fig 5d. No significant peak is observed in Si 2*p* and S 2*p* spectra.

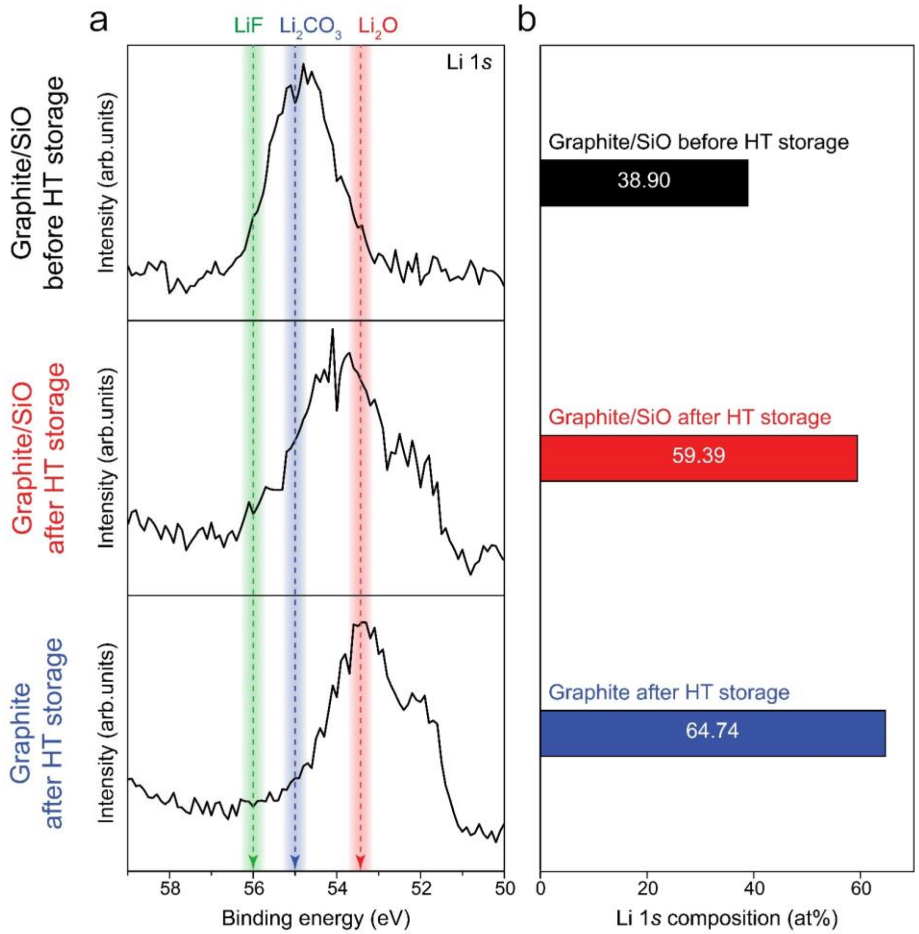

Supplementary Figure 15. XPS Li 1*s* spectra with corresponding elemental compositions of Li 1*s* when the components in all major elements (Li 1*s*, C 1*s*, O 1*s*, F 1*s*, and P 2*p*) were included in the calculation of relative composition.

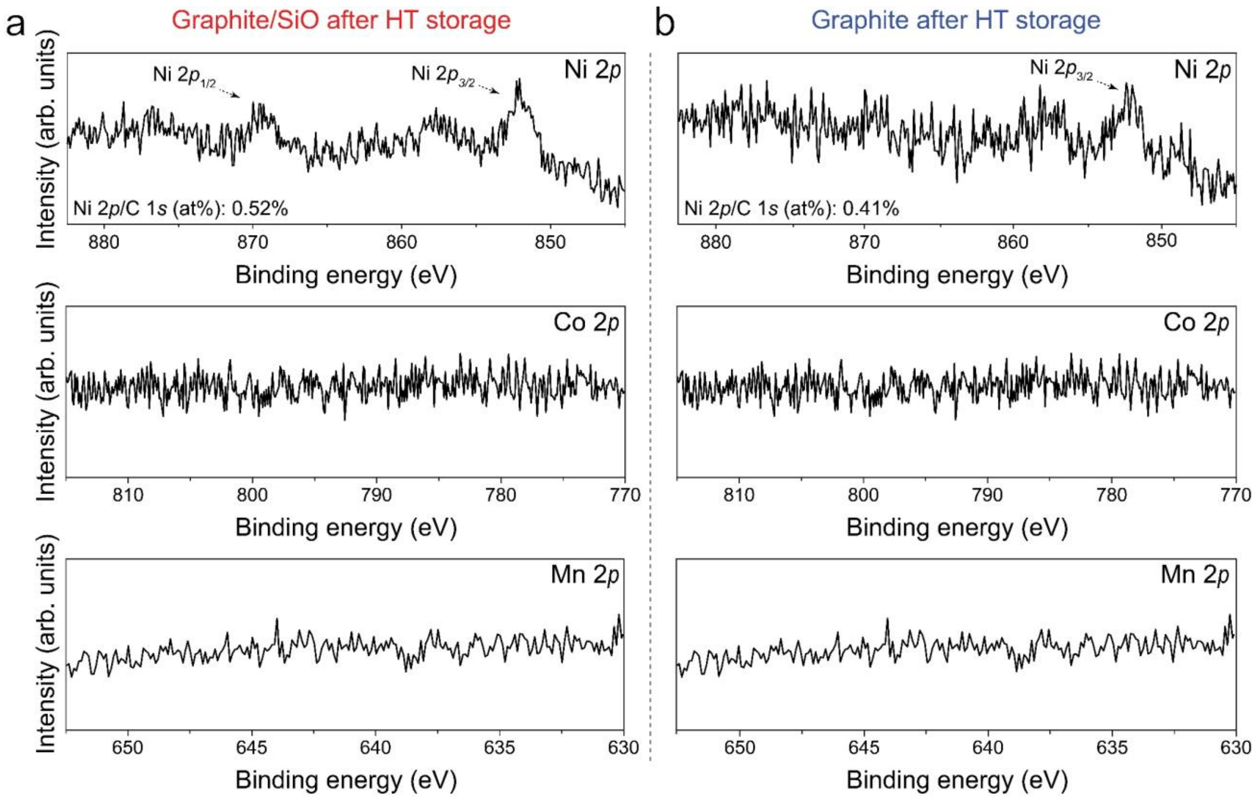

Supplementary Figure 16. XPS Ni 2*p*, Co 2*p*, and Mn 2*p* spectra of **a** graphite/SiO anode and **b** graphite/SiO after 4 weeks of HT storage. Presence of any peak in these spectra indicates that transition metal dissolution from NCM cathode affected the composition of SEI. A small amount of Ni was observed in Ni 2*p*, while no peaks were observed in Co 2*p* and Mn 2*p*.

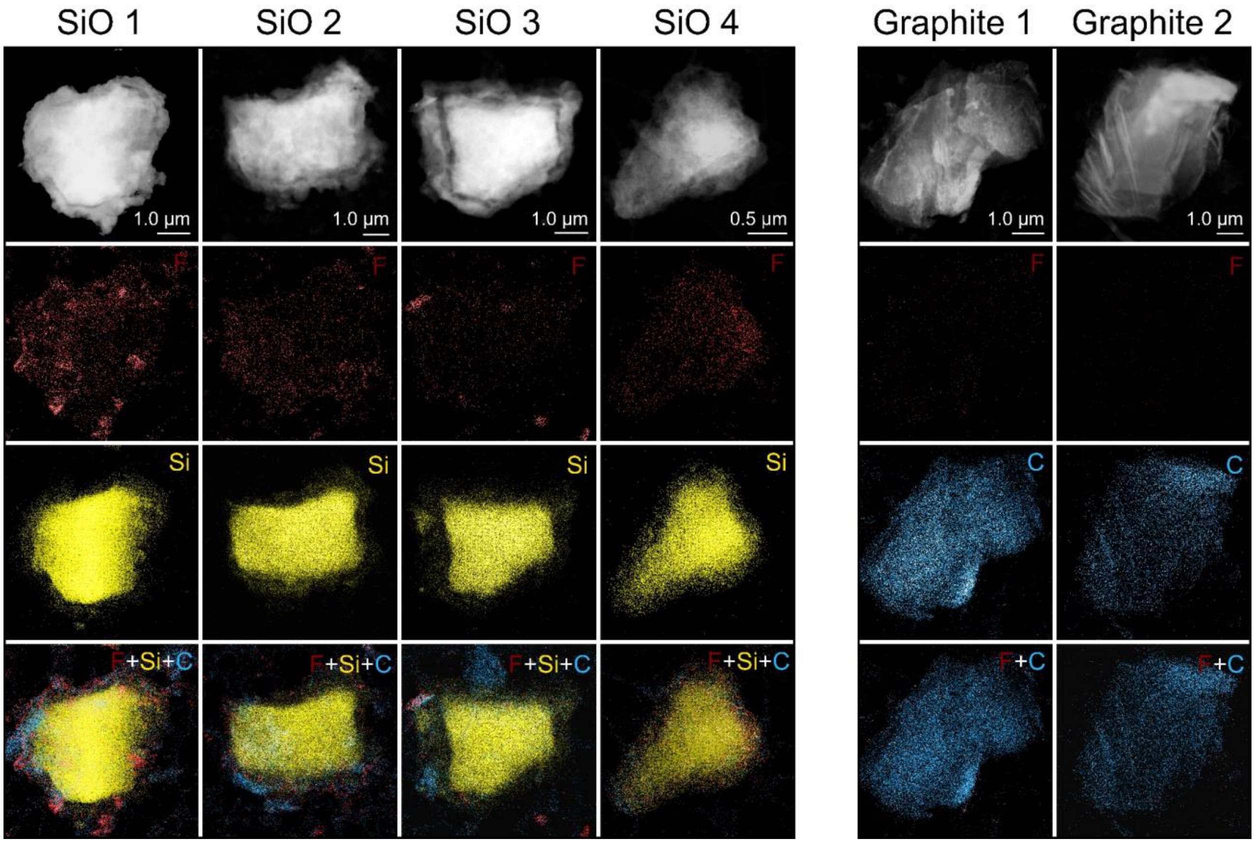

Supplementary Figure 17. TEM images and EDS elemental maps of SiO and graphite particles collected from graphite/SiO anode after 4 weeks of HT storage. Thicker SEI layers and larger amount of fluorine on SiO particles were observed compared to graphite particles.

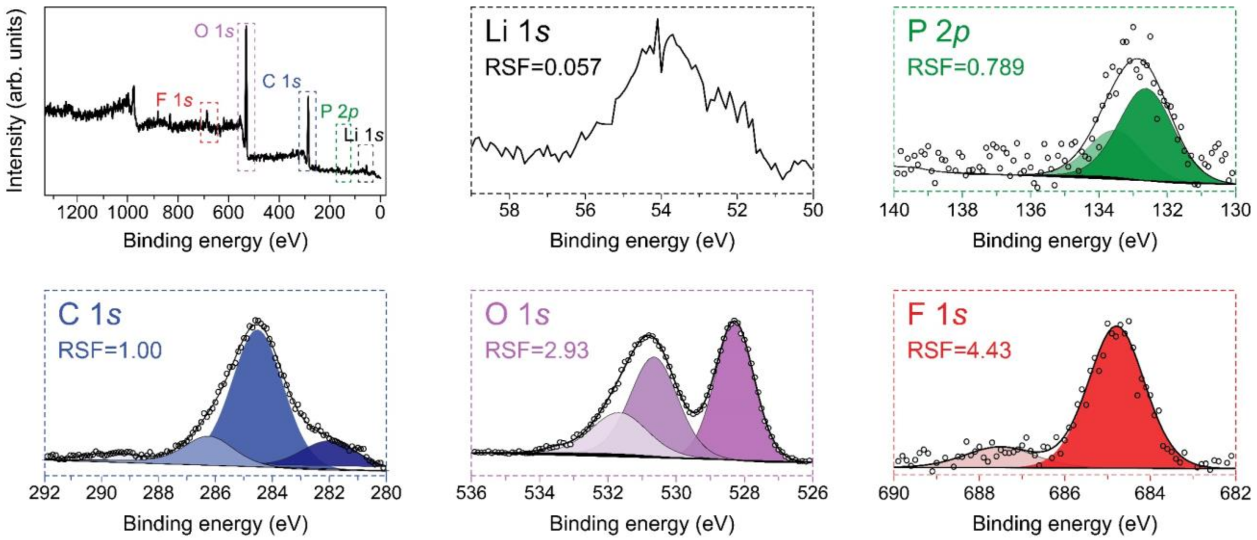

Supplementary Figure 18. XPS survey spectrum of graphite/SiO anode after HT storage and the narrow spectra of core elements with their RSFs used in quantifying elemental compositions of those in Fig. 5d and Supplementary Fig. 15b.

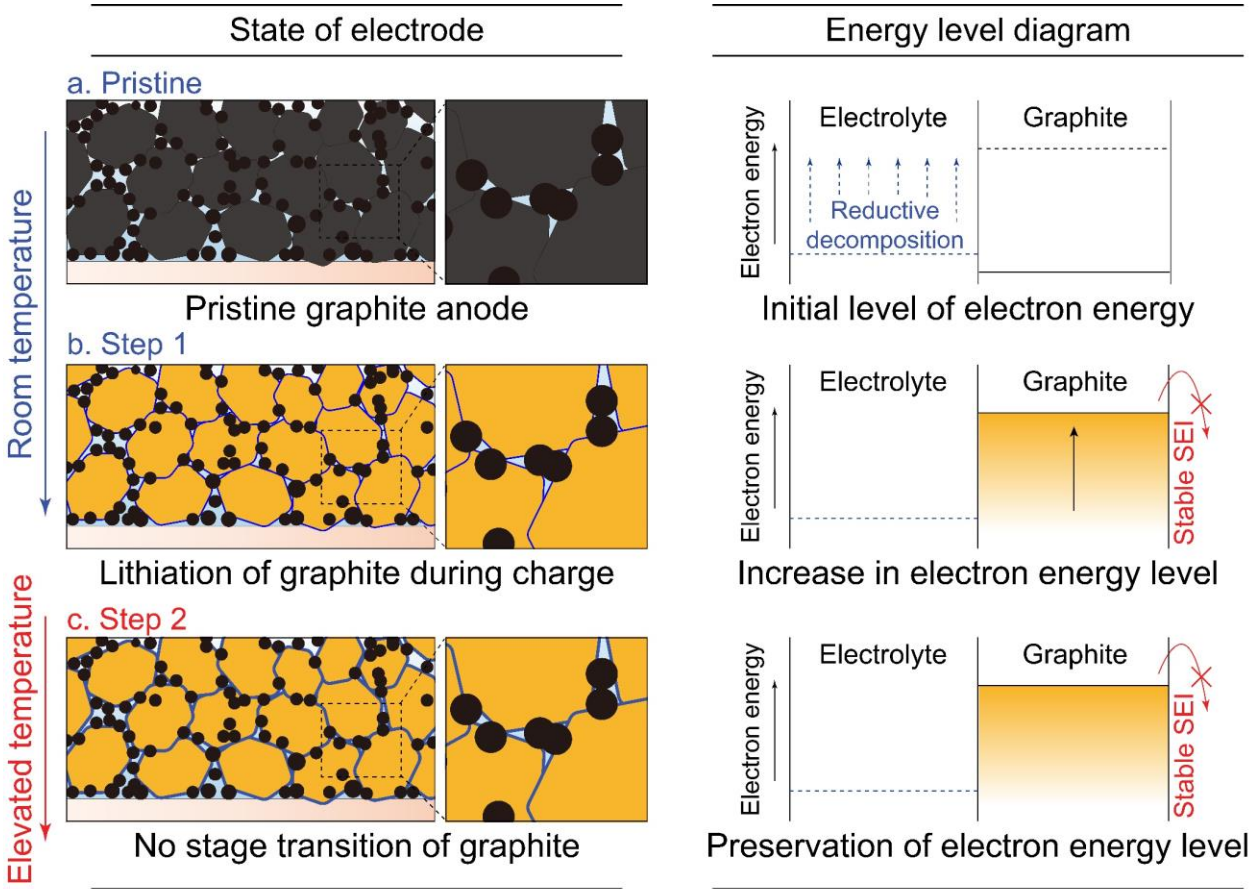

Supplementary Figure 19. Schematic procedures and relative energy diagrams for each state of graphite anode describing negligible thermal impact on charged graphite anode. **a.** Pristine state, fully wetted anode comprised of graphite (grey) and conductive agent (black). **b.** Step 1, fully lithiated graphite (yellow) after charging. **c.** Step 2, negligible de-lithiation of graphite by stable SEI preventing electron transfer effectively under the elevated temperature.

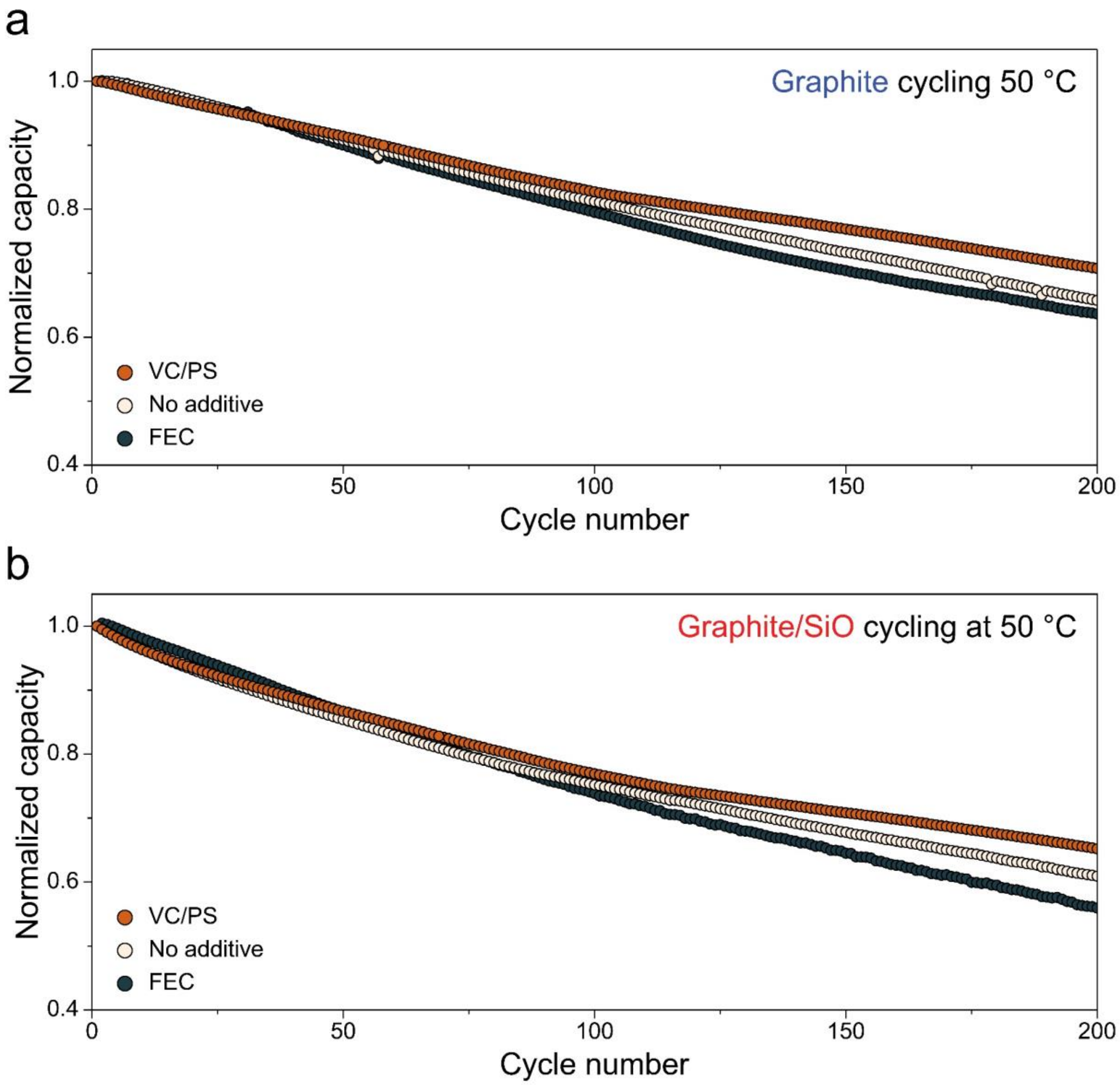

Supplementary Figure 20. Normalized discharge capacity of **a** graphite and **b** graphite/SiO full cell cycled at 50 ℃ with 1.0 M $LiPF_6$ in EC/EMC (3:7, vol%) with VC/PS (orange), no additive (white), 10 wt% FEC (navy).

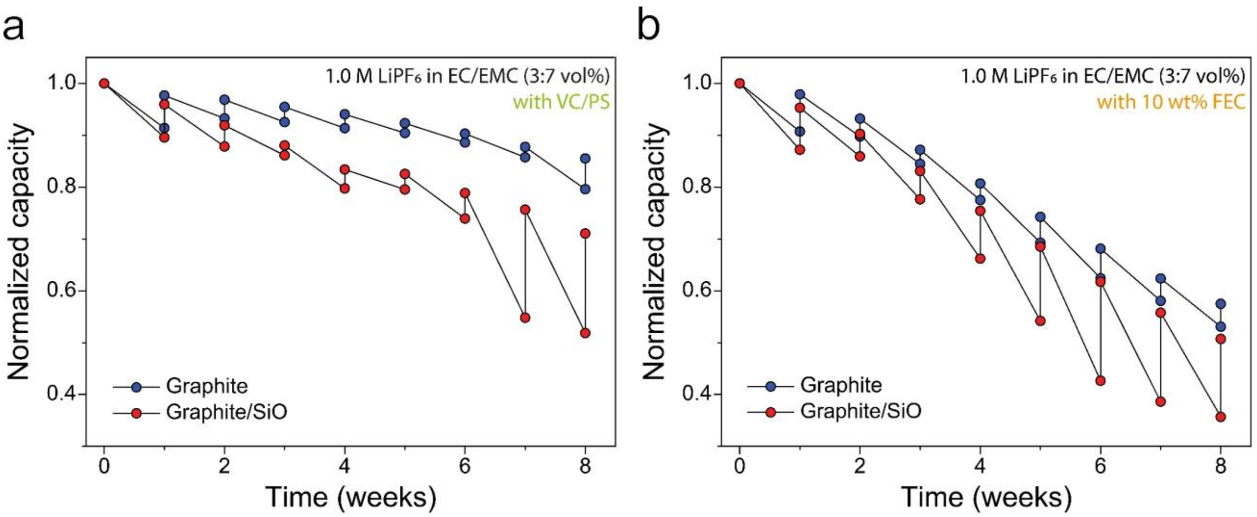

Supplementary Figure 21. Normalized retention and recovery capacities measured between periodic HT storage with **a** 1.0 M $LiPF_6$ in EC/EMC (3:7, vol%) with VC/PS (standard electrolyte) and **b** 1.0 M $LiPF_6$ in EC/EMC (3:7, vol%) with 10 wt% FEC.

Components		Graphite/SiO before HT storage (%)	Graphite/SiO after HT storage (%)	Graphite after HT storage (%)
C 1*s*	Li_xC	5.51	7.83	4.91
	C–C	36.10	35.32	28.85
	C–O	6.53	4.48	3.12
	$–CO_3$	5.40	1.95	0.91
	**C 1*s* total**	**53.54**	**49.58**	**37.79**
O 1*s*	Li_2O	12.80	19.85	34.75
	ROLi	13.30	18.13	20.26
	C=O	18.70	7.44	3.94
	**O 1*s* total**	**44.80**	**45.42**	**58.95**
F 1*s*	LiF	1.65	2.22	1.56
	$Li_xPF_yO_z$	0.00	0.39	0.00
	**F 1*s* total**	**1.65**	**2.61**	**1.56**
P 2*p*	P–O	0.00	2.39	1.70
	P–F	0.00	0.00	0.00
	**P 2*p* total**	**0.00**	**2.39**	**1.70**

Supplementary Table 1. Relative quantitative compositions of XPS C 1*s*, O 1*s*, F 1*s*, and P 2*p* spectra of graphite/SiO anode before HT storage, graphite/SiO and graphite anode after 4 weeks of HT storage.

Supplementary References

1. Kitada, K. *et al.* Unraveling the reaction mechanisms of SiO anodes for Li-ion batteries by combining *in situ* ^{7}Li and *ex situ* $^{7}Li/^{29}Si$ solid-state NMR spectroscopy. *J. Am. Chem. Soc*. **141**, 7014–7027 (2019).

2. Ozawa, Y., Yazami, R. & Fultz, B. Self-discharge study of $LiCoO_2$ cathode materials. *J. Power Sources* **119–121**, 918–923 (2003).

3. Seok, J., Gannett, C. N., Yu, S.-H. & Abruña, H. D. Understanding the impacts of Li stripping overpotentials at the counter electrode by three-electrode coin cell measurements. *Anal. Chem.* **93**, 15459–15467 (2021).

4. Schmitt, J., Schindler, M., Oberbauer, A. & Jossen. A. Determination of degradation modes of lithium-ion batteries considering aging-induced changes in the half-cell open-circuit potential curve of silicon–graphite. *J. Power Sources* **532**, 231296478 (2022).

5. Birkl, C. R. *et al.* Degradation diagnostics for lithium-ion cells. *J. Power Sources* **341**, 373–386 (2017).

6. Klett, M. *et al.* Electrode behavior re-visited: monitoring potential windows, capacity loss, and impedance changes in $Li_{1.03}(Ni_{0.5}Co_{0.2}Mn_{0.3})_{0.97}O_2$/silicon-graphite full cells. *J. Electrochem. Soc.* **163**, A875–A887 (2016).
